# Supplementary material for: Linking glycemic dysregulation in diabetes to symptoms, comorbidities, and genetics through EHR data mining
Source: eLife. 2019 Dec 10;8:e44941. doi: 10.7554/eLife.44941 (PMC6904221; doi:10.7554/eLife.44941)
Supplement: Supplementary file 1. [file elife-44941-supp1.docx]

**Supplementary Materials**

**Kirk and Simon et al.,**

**Linking glycemic dysregulation in diabetes to symptoms, comorbidities and genetics through EHR data mining.**

**Supplementary Table 1. Statistics for the metadata.** Mean, standard deviation and p-value for metadata for each of the 71 clusters with at least 50 individuals.

| **Cluster** | **Symptom Group** | **N** | **Sex (% males)** | **Primary diabetes type (count)** | | | | | **Age at last SDC visit (years)** | | **Diabetes duration (years)** | | **Time with diabetes prior to SDC (days)** | | **Contact with SDC (days)** | | **Primary (A) diagnoses** | | **Secondary (B) diagnoses** | |
| --- | --- | --- | --- | --- | --- | --- | --- | --- | --- | --- | --- | --- | --- | --- | --- | --- | --- | --- | --- | --- |
|  |  |  |  | **T1D** | **T2D** | **E13** | **E14** | **UnKn** | **Mean±sd** | **P-value** | **Mean±sd** | **P-value** | **Mean±sd** | **P-value** | **Mean±sd** | **P-value** | **Mean±sd** | **P-value** | **Mean±sd** | **P-value** |
| **1** | 5.1 | 979 | 68.9 | 541 | 433 | 3 | 0 | 2 | 61±15,8 | 0.0091 | 26±15,4 | 5.40E-38 | 2497±1497,5 | 1 | 3582±2388,5 | 5.50E-09 | 2±1 | 0.027 | 5±5 | 2.10E-63 |
| **2** | 3 | 399 | 68.2 | 266 | 126 | 1 | 4 | 2 | 46±17,1 | 5.00E-45 | 11±11,4 | 5.30E-46 | 2149±1379,4 | 0.039 | 2214±1935,1 | 5.00E-09 | 1±1,2 | 0.00018 | 1±1,8 | 8.00E-48 |
| **3** | 4 | 319 | 61.4 | 44 | 272 | 1 | 0 | 2 | 73±9,7 | 3.40E-56 | 17±12 | 1 | 2325±1356,9 | 1 | 2522±2125,2 | 0.018 | 2±0,9 | 1 | 4±2,7 | 2.00E-27 |
| **4** | 4 | 306 | 21.2 | 178 | 119 | 0 | 3 | 6 | 46±15,1 | 5.80E-38 | 19±13,2 | 1 | 2581±1477,1 | 1 | 3233±3112,2 | 1 | 3±1,9 | 2.10E-24 | 1±1,9 | 1.10E-19 |
| **5** | 4 | 276 | 68.8 | 93 | 182 | 0 | 1 | 0 | 60±13,3 | 1 | 22±12,2 | 0.0011 | 2593±1390,7 | 1 | 4157±2223,7 | 2.60E-12 | 2±0,8 | 1 | 4±2,4 | 9.20E-12 |
| **6** | 2 | 231 | 66.2 | 27 | 204 | 0 | 0 | 0 | 61±12,4 | 1 | 11±8,1 | 1.80E-20 | 2241±1300,3 | 1 | 1300±1740,3 | 1.30E-31 | 1±0,6 | 6.70E-07 | 2±1,5 | 1 |
| **7** | 4 | 225 | 43.1 | 108 | 115 | 0 | 0 | 2 | 54±16,4 | 0.013 | 17±13 | 0.045 | 2378±1383,9 | 1 | 2568±2455,1 | 0.072 | 2±1 | 1 | 2±1,6 | 2.90E-05 |
| **8** | 5.0 | 223 | 58.7 | 44 | 179 | 0 | 0 | 0 | 69±10,7 | 3.80E-19 | 19±12,9 | 1 | 2375±1350,9 | 1 | 2726±2203,6 | 1 | 1±0,7 | 1 | 4±2,6 | 1.10E-14 |
| **9** | 3 | 190 | 61.6 | 131 | 57 | 0 | 1 | 1 | 60±12,1 | 1 | 26±14,2 | 2.40E-08 | 2365±1511,8 | 1 | 4753±2312 | 1.70E-17 | 2±0,8 | 1 | 2±1,6 | 0.74 |
| **10** | 2 | 173 | 65.9 | 91 | 75 | 0 | 6 | 1 | 52±15,5 | 1.50E-05 | 15±11,3 | 0.0023 | 2369±1476,3 | 1 | 2400±2199 | 0.19 | 1±0,6 | 0.087 | 1±1,2 | 3.60E-24 |
| **11** | 5.2 | 168 | 74.4 | 94 | 60 | 11 | 2 | 1 | 52±15,9 | 4.70E-05 | 18±12,1 | 1 | 2472±1378 | 1 | 2916±2447 | 1 | 1±0,9 | 1 | 2±2,1 | 2.30E-07 |
| **12** | 5.2 | 155 | 41.3 | 70 | 82 | 1 | 0 | 2 | 55±17,4 | 1 | 17±13,4 | 0.35 | 2434±1461,2 | 1 | 2859±2391,5 | 1 | 2±0,9 | 1 | 2±2 | 1 |
| **13** | 4 | 151 | 43.7 | 64 | 85 | 0 | 1 | 1 | 51±16,7 | 5.00E-05 | 15±12,5 | 0.00035 | 2295±1348,5 | 1 | 2160±2195,2 | 0.0024 | 1±0,7 | 1 | 1±1,6 | 3.90E-12 |
| **14** | 2 | 145 | 71.7 | 119 | 26 | 0 | 0 | 0 | 51±14,9 | 2.00E-07 | 22±12,3 | 1 | 2619±1392,7 | 1 | 4029±2395,7 | 0.00014 | 2±0,8 | 1 | 1±1,8 | 1.10E-14 |
| **15** | 5.1 | 133 | 45.1 | 81 | 51 | 1 | 0 | 0 | 57±13,3 | 1 | 26±15,9 | 0.0089 | 2472±1587,2 | 1 | 4016±2610,7 | 0.001 | 2±1,1 | 1 | 2±2,1 | 1 |
| **16** | 4 | 127 | 54.3 | 40 | 82 | 3 | 0 | 2 | 61±13,6 | 1 | 18±14 | 1 | 2351±1445,9 | 1 | 2759±2463,3 | 1 | 1±0,8 | 1 | 3±2,5 | 1 |
| **17** | 4 | 124 | 38.7 | 56 | 66 | 0 | 2 | 0 | 60±16 | 1 | 21±14,5 | 1 | 2439±1403,9 | 1 | 3060±2464,2 | 1 | 2±1,1 | 1 | 3±2,1 | 1 |
| **18** | 4 | 124 | 53.2 | 48 | 74 | 0 | 1 | 1 | 64±14,9 | 0.0045 | 23±13,7 | 0.13 | 2540±1394,2 | 1 | 3692±2429,4 | 0.88 | 2±1 | 1 | 4±2,7 | 0.00073 |
| **19** | 5.2 | 120 | 51.7 | 56 | 63 | 0 | 0 | 1 | 67±13,3 | 1.20E-06 | 26±17,6 | 0.14 | 2115±1466 | 1 | 3262±2456,9 | 1 | 1±0,8 | 1 | 3±2,5 | 1 |
| **20** | 4 | 117 | 43.6 | 50 | 64 | 0 | 1 | 2 | 61±13,9 | 1 | 22±15,4 | 1 | 2504±1478 | 1 | 3407±2644,3 | 1 | 2±0,7 | 1 | 2±2 | 1 |
| **21** | 5.1 | 114 | 62.3 | 31 | 80 | 1 | 1 | 1 | 68±11,2 | 8.60E-09 | 22±12,5 | 1 | 2368±1307 | 1 | 4036±2222,7 | 0.0045 | 2±0,9 | 1 | 5±4,1 | 1.20E-11 |
| **22** | 5.0 | 113 | 52.2 | 25 | 88 | 0 | 0 | 0 | 69±12,6 | 3.70E-11 | 23±14,4 | 1 | 2558±1397,5 | 1 | 3439±2211,9 | 1 | 2±0,7 | 1 | 4±2,5 | 4.70E-09 |
| **23** | 5.1 | 110 | 73.6 | 44 | 65 | 0 | 1 | 0 | 60±14,7 | 1 | 22±13,4 | 1 | 2265±1317,4 | 1 | 3616±2332,3 | 1 | 2±0,9 | 1 | 4±3,5 | 1 |
| **24** | 4 | 108 | 58.3 | 69 | 38 | 0 | 0 | 1 | 51±16,8 | 0.0081 | 23±14,1 | 1 | 2753±1452,2 | 1 | 3791±2476,9 | 0.33 | 2±0,9 | 1 | 4±3,3 | 0.32 |
| **25** | 5.2 | 106 | 71.7 | 15 | 14 | 76 | 0 | 1 | 61±13,1 | 1 | 16±8,6 | 1 | 2172±1283,6 | 1 | 3489±2123,5 | 1 | 2±0,9 | 1 | 3±2,7 | 1 |
| **26** | 5.0 | 105 | 43.8 | 30 | 74 | 1 | 0 | 0 | 70±10,9 | 1.60E-12 | 24±15,4 | 0.6 | 2196±1453 | 1 | 3886±2463,6 | 0.3 | 2±1,2 | 0.067 | 4±2,9 | 4.60E-05 |
| **27** | 4 | 104 | 46.2 | 36 | 68 | 0 | 0 | 0 | 64±15 | 0.56 | 20±15,2 | 1 | 2248±1497,7 | 1 | 2926±2487,2 | 1 | 2±0,9 | 1 | 3±2,5 | 1 |
| **28** | 5.2 | 103 | 55.3 | 38 | 63 | 0 | 1 | 1 | 50±17,2 | 4.00E-04 | 11±9,6 | 3.80E-09 | 2345±1486,5 | 1 | 1535±1983 | 5.70E-10 | 1±0,6 | 0.0023 | 1±1,6 | 2.00E-10 |
| **29** | 5.2 | 102 | 86.3 | 56 | 45 | 1 | 0 | 0 | 59±15 | 1 | 22±13,4 | 1 | 2443±1454,5 | 1 | 3743±2387,6 | 0.77 | 2±1,3 | 1 | 2±1,8 | 0.034 |
| **30** | 4 | 99 | 13.1 | 65 | 33 | 0 | 0 | 1 | 42±13,4 | 1.40E-19 | 17±11,1 | 1 | 2657±1344,2 | 1 | 2560±2317,1 | 1 | 2±1,4 | 1 | 2±2,6 | 1 |
| **31** | 5.0 | 100 | 36 | 62 | 38 | 0 | 0 | 0 | 58±12,6 | 1 | 29±13,9 | 6.70E-10 | 2455±1520,3 | 1 | 4851±2082,8 | 2.20E-10 | 2±0,9 | 1 | 3±2,4 | 1 |
| **32** | 5.2 | 97 | 33 | 83 | 14 | 0 | 0 | 0 | 44±15,9 | 2.70E-14 | 23±13,4 | 1 | 2355±1472,8 | 1 | 3572±2458,1 | 1 | 2±1,2 | 1 | 2±2,2 | 0.27 |
| **33** | 5.0 | 96 | 49 | 25 | 69 | 0 | 0 | 2 | 62±14,2 | 1 | 18±13,2 | 1 | 2246±1441,6 | 1 | 2845±2509,7 | 1 | 2±0,9 | 1 | 2±1,9 | 1 |
| **34** | 4 | 96 | 78.1 | 16 | 79 | 0 | 0 | 1 | 59±9,9 | 1 | 14±11,9 | 0.0064 | 2414±1344,4 | 1 | 2039±2298,9 | 0.00084 | 2±0,7 | 1 | 3±2,5 | 1 |
| **35** | 5.0 | 93 | 73.1 | 20 | 70 | 0 | 0 | 3 | 66±11,3 | 0.004 | 18±14,7 | 1 | 2414±1405,6 | 1 | 2970±2486 | 1 | 1±0,8 | 1 | 4±2,4 | 0.00032 |
| **36** | 4 | 93 | 23.7 | 48 | 41 | 1 | 1 | 2 | 50±14,8 | 5.50E-05 | 15±11,8 | 0.41 | 2354±1494 | 1 | 2665±2316,2 | 1 | 2±1 | 1 | 2±1,9 | 0.0041 |
| **37** | 3 | 90 | 54.4 | 24 | 65 | 0 | 1 | 0 | 55±15,7 | 1 | 12±8,5 | 7.60E-05 | 2042±1415,4 | 1 | 2814±1766 | 1 | 1±0,6 | 1 | 2±2,3 | 1 |
| **38** | 3 | 90 | 68.9 | 31 | 58 | 0 | 0 | 1 | 53±16,7 | 0.31 | 13±10,2 | 0.0023 | 2466±1449 | 1 | 1514±1855,7 | 4.30E-08 | 1±0,7 | 1 | 1±2,1 | 7.00E-05 |
| **39** | 4 | 90 | 54.4 | 53 | 36 | 0 | 1 | 0 | 59±18,9 | 1 | 23±15,8 | 1 | 2288±1513,6 | 1 | 3248±2213,3 | 1 | 2±1,2 | 1 | 3±2,4 | 1 |
| **40** | 5.0 | 89 | 46.1 | 51 | 37 | 0 | 1 | 0 | 59±11,6 | 1 | 25±14,8 | 0.072 | 2420±1547,6 | 1 | 4224±2531 | 0.0025 | 2±1 | 1 | 2±1,9 | 1 |
| **41** | 5.2 | 84 | 69 | 78 | 5 | 1 | 0 | 0 | 53±15,9 | 0.16 | 29±13,7 | 3.90E-07 | 2239±1594,6 | 1 | 4716±2300,3 | 1.80E-07 | 2±0,9 | 0.51 | 2±2,1 | 1 |
| **42** | 5.1 | 84 | 59.5 | 61 | 21 | 2 | 0 | 0 | 52±9,7 | 0.002 | 33±13,8 | 4.40E-13 | 2711±1835 | 1 | 4471±2424,3 | 0.00018 | 1±1,2 | 1 | 6±4,3 | 4.80E-16 |
| **43** | 5.1 | 83 | 67.5 | 43 | 38 | 1 | 0 | 1 | 57±16,5 | 1 | 19±13,8 | 1 | 2403±1350,8 | 1 | 3136±2443,8 | 1 | 2±1,1 | 1 | 3±2,5 | 1 |
| **44** | 2 | 80 | 56.2 | 48 | 30 | 2 | 0 | 0 | 49±17,2 | 0.00016 | 15±11,6 | 0.6 | 2212±1390,2 | 1 | 2423±2273,4 | 1 | 1±0,6 | 0.091 | 1±1,1 | 3.40E-15 |
| **45** | 5.0 | 80 | 67.5 | 8 | 72 | 0 | 0 | 0 | 72±9,4 | 7.20E-12 | 18±9,8 | 1 | 2599±1236 | 1 | 2894±2048,2 | 1 | 1±0,7 | 1 | 5±3,6 | 8.90E-12 |
| **46** | 4 | 79 | 45.6 | 39 | 40 | 0 | 0 | 0 | 63±16,9 | 1 | 22±15,7 | 1 | 2411±1430,9 | 1 | 3133±2453 | 1 | 1±0,7 | 1 | 2±2,2 | 1 |
| **47** | 4 | 77 | 58.4 | 28 | 48 | 1 | 0 | 0 | 49±13,7 | 2.40E-05 | 16±12,3 | 1 | 2458±1552,4 | 1 | 2847±2385,2 | 1 | 2±1 | 1 | 3±2,7 | 1 |
| **48** | 3 | 75 | 64 | 21 | 52 | 0 | 1 | 1 | 63±14,2 | 1 | 17±13,1 | 1 | 2067±1384,8 | 1 | 2529±2370,1 | 1 | 1±0,8 | 1 | 2±2,5 | 1 |
| **49** | 5.2 | 75 | 66.7 | 71 | 4 | 0 | 0 | 0 | 41±18,2 | 7.00E-13 | 23±11,2 | 0.56 | 2666±1424,4 | 1 | 3517±2489,9 | 1 | 1±0,6 | 1 | 1±2 | 5.50E-07 |
| **50** | 3 | 69 | 85.5 | 9 | 60 | 0 | 0 | 0 | 61±11,7 | 1 | 13±9,2 | 0.0059 | 2276±1422,7 | 1 | 1604±1605,1 | 4.30E-05 | 1±0,7 | 1 | 2±1,7 | 1 |
| **51** | 5.2 | 67 | 44.8 | 34 | 33 | 0 | 0 | 0 | 56±12,1 | 1 | 20±13,9 | 1 | 2261±1443,2 | 1 | 3483±2611,4 | 1 | 2±0,8 | 1 | 2±1,9 | 1 |
| **52** | 5.2 | 64 | 73.4 | 20 | 42 | 0 | 0 | 2 | 61±17,4 | 1 | 14±10,5 | 0.057 | 2362±1279,6 | 1 | 1644±1922,7 | 0.00022 | 1±0,8 | 1 | 2±2,3 | 0.35 |
| **53** | 5.1 | 63 | 66.7 | 49 | 11 | 0 | 0 | 3 | 58±10,3 | 1 | 36±15,7 | 6.70E-11 | 2804±1704,1 | 1 | 5391±2140,1 | 2.30E-11 | 2±1 | 1 | 2±2,2 | 1 |
| **54** | 3 | 61 | 54.1 | 23 | 38 | 0 | 0 | 0 | 55±15,5 | 1 | 17±13,7 | 1 | 2086±1306,4 | 1 | 2479±2324 | 1 | 2±0,8 | 1 | 2±1,8 | 1 |
| **55** | 5.0 | 60 | 63.3 | 22 | 37 | 1 | 0 | 0 | 52±14 | 0.18 | 15±8,3 | 1 | 2064±1200,7 | 1 | 2480±2458,2 | 1 | 2±1,1 | 1 | 2±2,3 | 0.071 |
| **56** | 4 | 59 | 98.3 | 10 | 49 | 0 | 0 | 0 | 73±8,1 | 5.30E-11 | 19±13,5 | 1 | 2173±1354,5 | 1 | 2453±2337,7 | 1 | 2±0,8 | 1 | 3±2,6 | 1 |
| **57** | 5.2 | 60 | 61.7 | 36 | 23 | 0 | 0 | 1 | 54±16,6 | 1 | 17±10,1 | 1 | 2644±1258,8 | 1 | 3439±2526,8 | 1 | 2±0,8 | 1 | 1±1,8 | 0.0066 |
| **58** | 2 | 56 | 64.3 | 25 | 18 | 0 | 0 | 13 | 57±11,7 | 1 | 25±14,8 | 1 | 3247±1569,9 | 0.0023 | 3293±2476,9 | 1 | 4±3,4 | 1.30E-07 | 1±1,4 | 0.00012 |
| **59** | 5.0 | 56 | 30.4 | 18 | 35 | 0 | 0 | 3 | 64±19,4 | 1 | 17±11,2 | 1 | 2398±1384,9 | 1 | 2815±2564,7 | 1 | 2±0,9 | 1 | 2±2,2 | 1 |
| **60** | 5.2 | 56 | 48.2 | 38 | 18 | 0 | 0 | 0 | 62±19,4 | 1 | 26±15 | 0.32 | 2663±1525,1 | 1 | 4048±2380,3 | 0.31 | 2±0,8 | 1 | 2±1,8 | 1 |
| **61** | 5.0 | 55 | 92.7 | 14 | 38 | 2 | 0 | 1 | 74±8 | 2.80E-12 | 18±11,9 | 1 | 2389±1454,4 | 1 | 3053±2199,5 | 1 | 2±0,8 | 1 | 3±3,5 | 1 |
| **62** | 5.2 | 55 | 58.2 | 17 | 36 | 1 | 1 | 0 | 60±13,3 | 1 | 18±13,4 | 1 | 2144±1413,8 | 1 | 2874±2287,1 | 1 | 2±0,8 | 1 | 3±2 | 1 |
| **63** | 3 | 54 | 72.2 | 20 | 31 | 2 | 0 | 1 | 59±14,4 | 1 | 20±13,2 | 1 | 2497±1370,9 | 1 | 3308±2167,3 | 1 | 2±0,8 | 1 | 2±1,9 | 1 |
| **64** | 5.2 | 54 | 66.7 | 18 | 36 | 0 | 0 | 0 | 54±15,2 | 1 | 14±9,3 | 0.66 | 2653±1386,5 | 1 | 1417±1903,3 | 1.70E-06 | 1±0,6 | 1 | 1±1,6 | 0.036 |
| **65** | 5.2 | 53 | 49.1 | 8 | 41 | 1 | 1 | 2 | 63±12,9 | 1 | 18±10,8 | 1 | 2239±1346,4 | 1 | 2971±2305,3 | 1 | 2±0,8 | 1 | 3±2 | 1 |
| **66** | 4 | 53 | 56.6 | 26 | 26 | 1 | 0 | 0 | 64±15,4 | 1 | 26±12,2 | 0.013 | 2541±1535,3 | 1 | 4405±2231,7 | 0.02 | 2±1,3 | 1 | 4±3,8 | 0.025 |
| **67** | 4 | 52 | 13.5 | 28 | 22 | 0 | 0 | 2 | 58±11,6 | 1 | 25±16 | 1 | 2557±1402 | 1 | 3859±2601,3 | 1 | 2±0,9 | 1 | 3±2,2 | 1 |
| **68** | 4 | 52 | 46.2 | 27 | 24 | 0 | 0 | 1 | 62±15,2 | 1 | 25±17,1 | 1 | 2238±1444,8 | 1 | 3341±2501,9 | 1 | 1±0,8 | 1 | 3±2,6 | 1 |
| **69** | 5.2 | 52 | 67.3 | 20 | 30 | 0 | 0 | 2 | 55±15,6 | 1 | 13±8,5 | 0.2 | 2200±1455 | 1 | 2213±2203,9 | 1 | 1±1 | 1 | 2±1,7 | 1 |
| **70** | 1 | 51 | 68.6 | 36 | 13 | 0 | 0 | 2 | 36±14,8 | 8.10E-12 | 14±7,6 | 1 | 2609±1538,7 | 1 | 1919±2058,3 | 0.23 | 1±0,3 | 2.50E-05 | 0±0,1 | 2.00E-18 |
| **71** | 5.2 | 50 | 10 | 20 | 27 | 3 | 0 | 0 | 64±12,9 | 1 | 18±14 | 1 | 2334±1550 | 1 | 2631±2382,5 | 1 | 1±0,6 | 1 | 2±2 | 1 |
