# Supplementary material for: Linking glycemic dysregulation in diabetes to symptoms, comorbidities, and genetics through EHR data mining
Source: eLife. 2019 Dec 10;8:e44941. doi: 10.7554/eLife.44941 (PMC6904221; doi:10.7554/eLife.44941)
Supplement: Supplementary file 2. [file elife-44941-supp2.docx]

**Supplementary Materials**

**Kirk and Simon et al.,**

**Linking glycemic dysregulation in diabetes to symptoms, comorbidities and genetics through EHR data mining.**

**Supplementary Table 2. Statistics for the physiological test.** Mean, standard deviation and p-value for the biochemical test for each of the 71 clusters with at least 50 individuals.

| **Cluster** | **P/S Cholesterol+ester** | | **S proinsulin C-peptid** | | **P/S HDL Cholesterol** | | **P/S LDL Cholesterol** | | **P/S VLDL Cholesterol** | | **U-Creatinine** | | **Haemoglobin Beta (Iron)** | | **P/S Potassium** | | **P/S Sodium** | |
| --- | --- | --- | --- | --- | --- | --- | --- | --- | --- | --- | --- | --- | --- | --- | --- | --- | --- | --- |
|  | **Mean±sd** | **P-value** | **Mean±sd** | **P-value** | **Mean±sd** | **P-value** | **Mean±sd** | **P-value** | **Mean±sd** | **P-value** | **Mean±sd** | **P-value** | **Mean±sd** | **P-value** | **Mean±sd** | **P-value** | **Mean±sd** | **P-value** |
| **1** | 4,91±1,2 | 2.20E-05 | 687,21±711,5 | 0.00025 | 1,46±0,6 | 0.0085 | 2,59±1 | 5.50E-05 | 0,71±0,4 | 0.75 | 8296,57±5283,4 | 0.055 | 8,41±1 | 1.70E-10 | 4,25±0,5 | 6.00E-89 | 139,06±3,2 | 0.024 |
| **2** | 4,82±1,1 | 0.85 | 587,17±666 | 8.70E-08 | 1,49±0,5 | 0.28 | 2,56±0,9 | 0.031 | 0,69±0,4 | 0.11 | 10172,11±6259,6 | 4.00E-47 | 8,74±0,9 | 3.30E-32 | 4,09±0,4 | 2.80E-13 | 138,46±3,4 | 7.70E-18 |
| **3** | 4,75±1,2 | 0.00035 | 1160,5±914,4 | 1.90E-26 | 1,29±0,5 | 8.30E-58 | 2,45±1 | 0.00021 | 0,81±0,4 | 7.10E-26 | 7246,82±3760,9 | 1.30E-12 | 8,42±1 | 7.00E-04 | 4,19±0,5 | 0.00012 | 139,07±3,1 | 0.38 |
| **4** | 4,84±1,1 | 0.93 | 752,4±767,9 | 0.48 | 1,57±0,5 | 4.70E-25 | 2,54±0,8 | 0.3 | 0,65±0,4 | 4.00E-17 | 8607,38±5070,4 | 2.00E-04 | 8,21±0,8 | 1.50E-53 | 4,06±0,4 | 1.10E-34 | 138,63±2,9 | 2.70E-19 |
| **5** | 4,6±1,1 | 6.00E-42 | 919,94±844,9 | 0.0012 | 1,33±0,4 | 2.00E-58 | 2,36±0,9 | 5.20E-27 | 0,77±0,4 | 1.70E-23 | 7817,09±4502,1 | 3.30E-05 | 8,44±1 | 0.00038 | 4,14±0,4 | 0.0046 | 139,43±2,9 | 1.30E-06 |
| **6** | 5±1,2 | 0.00036 | 934,57±592,7 | 3.10E-07 | 1,28±0,4 | 1.40E-24 | 2,77±1 | 1.30E-09 | 0,81±0,5 | 5.10E-10 | 8409,44±5366,7 | 0.95 | 8,82±0,9 | 5.90E-17 | 4,15±0,4 | 0.92 | 139,77±2,7 | 3.90E-06 |
| **7** | 4,76±1,1 | 0.015 | 755,95±788,7 | 0.95 | 1,5±0,5 | 0.00031 | 2,52±0,9 | 0.54 | 0,69±0,4 | 0.0031 | 9100,17±5640 | 4.20E-05 | 8,48±0,8 | 0.22 | 4,09±0,4 | 3.70E-09 | 139,36±2,8 | 0.084 |
| **8** | 4,76±1,2 | 0.016 | 986,36±671,6 | 2.00E-10 | 1,33±0,5 | 9.70E-24 | 2,4±1 | 1.10E-06 | 0,85±0,5 | 9.00E-26 | 7632,01±4444,1 | 0.00023 | 8,53±0,9 | 0.53 | 4,22±0,4 | 2.00E-06 | 139,53±2,8 | 0.00017 |
| **9** | 4,99±1 | 8.30E-10 | 521,9±551,5 | 0.00017 | 1,73±0,6 | 7.90E-69 | 2,65±0,8 | 2.70E-09 | 0,55±0,3 | 1.40E-52 | 8668,56±5455,2 | 0.096 | 8,88±0,8 | 2.60E-46 | 4,09±0,4 | 1.20E-09 | 139,32±3 | 0.035 |
| **10** | 4,84±1 | 0.52 | 591,5±564,6 | 0.013 | 1,56±0,6 | 0.0046 | 2,59±0,9 | 0.026 | 0,63±0,4 | 1.70E-05 | 10029,14±5617,9 | 2.30E-24 | 8,91±0,8 | 3.30E-29 | 4,08±0,4 | 3.80E-07 | 139,72±2,7 | 3.00E-05 |
| **11** | 4,72±1,1 | 0.0019 | 540,59±572,9 | 0.00037 | 1,56±0,6 | 2.40E-07 | 2,48±0,9 | 0.14 | 0,64±0,4 | 1.20E-05 | 9732,78±5792,2 | 1.80E-13 | 9±0,9 | 6.70E-51 | 4,05±0,4 | 5.00E-13 | 139,19±3,3 | 0.4 |
| **12** | 4,67±1,1 | 2.80E-05 | 733,84±698 | 0.89 | 1,52±0,5 | 0.00023 | 2,38±0,9 | 2.70E-06 | 0,66±0,4 | 0.0033 | 8539,89±5682,9 | 0.74 | 8,59±0,9 | 0.0015 | 4,14±0,4 | 0.19 | 139,45±2,8 | 0.045 |
| **13** | 4,78±1 | 0.67 | 772,5±690,4 | 0.5 | 1,45±0,5 | 1 | 2,58±0,8 | 0.036 | 0,72±0,4 | 0.71 | 9044,65±5852,6 | 0.0062 | 8,68±0,8 | 2.50E-06 | 4,13±0,4 | 0.14 | 139,35±2,7 | 0.36 |
| **14** | 4,85±1 | 0.42 | 352,22±459,5 | 3.20E-15 | 1,6±0,5 | 2.20E-18 | 2,61±0,8 | 0.0032 | 0,58±0,4 | 9.40E-21 | 10246,19±5994,8 | 2.90E-27 | 9,04±0,7 | 3.50E-69 | 4,13±0,4 | 0.037 | 139,53±2,6 | 0.0038 |
| **15** | 4,84±1 | 0.42 | 750,65±817,1 | 0.43 | 1,56±0,5 | 2.50E-11 | 2,57±0,9 | 0.098 | 0,63±0,4 | 6.10E-09 | 8394,94±5209,2 | 0.95 | 8,62±0,8 | 0.00027 | 4,09±0,4 | 1.50E-06 | 139,26±2,8 | 0.94 |
| **16** | 5,01±1,3 | 0.012 | 917,6±765,7 | 0.0063 | 1,45±0,6 | 0.00016 | 2,61±1 | 0.31 | 0,8±0,4 | 1.10E-10 | 8093,9±5021,3 | 0.18 | 8,5±1 | 0.81 | 4,19±0,4 | 0.014 | 138,95±2,9 | 0.032 |
| **17** | 4,88±1,2 | 0.32 | 636,73±663,4 | 0.042 | 1,56±0,5 | 2.00E-08 | 2,45±1 | 0.0058 | 0,72±0,4 | 0.099 | 7340,36±4921,9 | 3.30E-12 | 8,39±0,9 | 0.00036 | 4,18±0,4 | 0.15 | 139,2±3 | 0.51 |
| **18** | 4,89±1,2 | 0.56 | 937,88±904,4 | 0.12 | 1,53±0,6 | 0.34 | 2,38±0,9 | 3.10E-06 | 0,79±0,4 | 7.50E-10 | 6550,65±4441,9 | 2.20E-37 | 7,95±1,1 | 6.30E-74 | 4,43±0,6 | 5.80E-112 | 137,74±3,8 | 7.70E-52 |
| **19** | 4,96±1,1 | 0.0022 | 786,88±986,9 | 0.72 | 1,61±0,6 | 1.20E-13 | 2,59±0,9 | 0.13 | 0,66±0,4 | 0.00011 | 7440,06±3993,3 | 0.0013 | 8,47±0,9 | 0.28 | 4,17±0,4 | 0.52 | 139,49±3 | 0.0017 |
| **20** | 4,96±1,1 | 0.00024 | 629,74±613 | 0.12 | 1,62±0,6 | 5.70E-14 | 2,55±1 | 0.84 | 0,66±0,4 | 0.0083 | 7036,45±4654,5 | 2.60E-16 | 8,44±0,9 | 0.16 | 4,14±0,5 | 0.057 | 139,52±2,9 | 0.0028 |
| **21** | 4,7±1,1 | 0.00015 | 1004,6±768,6 | 3.00E-06 | 1,4±0,5 | 5.10E-05 | 2,39±0,9 | 1.80E-05 | 0,77±0,4 | 3.40E-06 | 7032,92±4004 | 2.50E-14 | 8,35±1 | 8.90E-07 | 4,18±0,5 | 0.083 | 139,03±3,4 | 0.94 |
| **22** | 5,06±1,3 | 5.10E-06 | 788,46±599,9 | 0.21 | 1,36±0,5 | 4.20E-11 | 2,58±1 | 0.56 | 0,85±0,5 | 1.40E-16 | 7629,56±4683,4 | 0.00082 | 8,41±0,9 | 0.023 | 4,18±0,4 | 0.39 | 139,12±3,2 | 0.79 |
| **23** | 4,82±1,3 | 0.095 | 910,48±861 | 0.12 | 1,33±0,4 | 1.60E-16 | 2,45±0,9 | 0.0016 | 0,8±0,5 | 1.30E-08 | 8243,52±4950 | 0.79 | 8,48±0,9 | 0.77 | 4,24±0,4 | 2.70E-07 | 138,76±3,3 | 4.90E-05 |
| **24** | 4,9±1,3 | 0.51 | 559,04±747 | 3.00E-05 | 1,56±0,6 | 5.80E-07 | 2,53±1 | 0.87 | 0,67±0,4 | 3.80E-05 | 8260,21±5525 | 0.23 | 8,34±1 | 0.00028 | 4,17±0,5 | 0.84 | 138,54±3,7 | 1.20E-05 |
| **25** | 4,66±1,1 | 5.60E-05 | 537,02±746,2 | 0.001 | 1,54±0,5 | 0.0012 | 2,27±0,9 | 1.80E-09 | 0,71±0,4 | 0.64 | 7345,11±4705,9 | 8.70E-08 | 8,53±1 | 0.46 | 4,26±0,5 | 2.40E-07 | 138,17±3,4 | 2.70E-15 |
| **26** | 4,99±1,1 | 9.50E-05 | 985,07±784,9 | 0.0014 | 1,56±0,5 | 1.60E-07 | 2,52±1 | 0.71 | 0,76±0,4 | 0.00013 | 7163,78±4252,2 | 1.40E-10 | 8,23±0,9 | 8.00E-16 | 4,24±0,4 | 2.70E-08 | 138,93±3 | 0.012 |
| **27** | 5,07±1,1 | 9.20E-07 | 962,2±801,4 | 0.0073 | 1,47±0,6 | 0.37 | 2,67±0,9 | 0.00025 | 0,78±0,4 | 7.90E-05 | 7625,17±4399,9 | 0.0021 | 8,45±1 | 0.39 | 4,22±0,4 | 4.40E-05 | 138,7±3,4 | 0.0016 |
| **28** | 4,96±1,2 | 0.064 | 711,4±599,6 | 0.83 | 1,33±0,5 | 9.30E-06 | 2,65±1 | 0.032 | 0,84±0,5 | 2.70E-06 | 9853,52±5910 | 1.40E-08 | 8,93±1 | 4.60E-13 | 4,15±0,4 | 0.56 | 140,04±2,5 | 7.20E-07 |
| **29** | 4,78±1,1 | 0.62 | 832,17±1074,1 | 0.66 | 1,45±0,5 | 0.44 | 2,54±0,9 | 0.31 | 0,67±0,4 | 0.003 | 10616,81±6095,4 | 5.80E-28 | 8,8±0,8 | 6.80E-17 | 4,12±0,4 | 0.013 | 139,93±2,7 | 9.40E-11 |
| **30** | 4,9±1,1 | 0.12 | 443,94±628,8 | 4.50E-12 | 1,52±0,6 | 0.0064 | 2,59±0,8 | 0.048 | 0,71±0,4 | 0.6 | 8226,53±5692,1 | 0.028 | 8,17±0,8 | 1.00E-16 | 4,07±0,4 | 9.90E-09 | 138,42±3,2 | 2.30E-08 |
| **31** | 4,81±1 | 0.82 | 481,51±550,5 | 7.70E-06 | 1,57±0,5 | 1.20E-17 | 2,54±0,9 | 0.56 | 0,63±0,4 | 9.20E-09 | 8022,49±5127 | 0.026 | 8,58±0,9 | 0.025 | 4,12±0,4 | 0.018 | 139,39±2,8 | 0.024 |
| **32** | 4,83±1,2 | 0.094 | 203,49±316,1 | 2.20E-25 | 1,55±0,4 | 1.20E-08 | 2,62±1 | 0.34 | 0,57±0,4 | 2.50E-22 | 8360,57±5346,5 | 0.62 | 8,18±1 | 2.50E-14 | 4,05±0,4 | 7.30E-11 | 138,97±3 | 0.15 |
| **33** | 4,88±1,3 | 0.8 | 832,5±519,4 | 0.016 | 1,33±0,5 | 4.60E-10 | 2,54±0,9 | 0.76 | 0,74±0,4 | 0.023 | 8807,89±5531,8 | 0.055 | 8,5±1 | 0.48 | 4,15±0,4 | 0.71 | 139,34±2,8 | 0.27 |
| **34** | 4,54±1 | 9.20E-07 | 1177,43±850,4 | 6.10E-07 | 1,28±0,5 | 1.20E-19 | 2,41±0,9 | 0.048 | 0,8±0,4 | 3.40E-06 | 9267,79±5128,1 | 4.00E-10 | 8,67±0,8 | 0.00038 | 4,08±0,4 | 0.00012 | 139,58±2,8 | 0.02 |
| **35** | 4,82±1,3 | 0.32 | 1238,64±899,8 | 3.70E-10 | 1,29±0,5 | 3.50E-25 | 2,37±1 | 8.20E-06 | 0,91±0,5 | 3.00E-29 | 7669,96±4785,7 | 0.00073 | 8,39±0,9 | 0.0087 | 4,25±0,5 | 5.20E-12 | 139,38±3 | 0.1 |
| **36** | 4,71±1 | 0.032 | 656,83±731,1 | 0.17 | 1,4±0,4 | 0.13 | 2,54±0,9 | 0.83 | 0,7±0,4 | 1 | 9027,92±5768,5 | 0.085 | 8,54±0,8 | 0.8 | 4,03±0,4 | 2.20E-10 | 139,19±2,8 | 0.94 |
| **37** | 4,87±1,2 | 0.67 | 872,72±569,9 | 0.00018 | 1,27±0,4 | 1.20E-17 | 2,6±0,9 | 0.11 | 0,79±0,4 | 1.00E-06 | 8144,88±5676,4 | 0.025 | 8,66±1 | 0.00042 | 4,09±0,4 | 5.50E-05 | 139,58±2,7 | 0.0051 |
| **38** | 4,42±1,1 | 1.50E-10 | 861,11±834,5 | 0.41 | 1,26±0,4 | 3.00E-14 | 2,29±0,9 | 3.10E-06 | 0,83±0,4 | 8.20E-09 | 9755,47±5769 | 1.20E-07 | 8,98±0,7 | 3.10E-20 | 4,02±0,4 | 5.70E-08 | 140,04±2,6 | 2.20E-06 |
| **39** | 4,87±1,2 | 0.93 | 599,72±654,2 | 0.02 | 1,4±0,5 | 3.70E-05 | 2,48±0,9 | 0.2 | 0,81±0,4 | 3.10E-13 | 7184,44±4859,4 | 3.90E-10 | 8,24±1 | 3.10E-11 | 4,25±0,5 | 1.70E-09 | 138,07±3,5 | 4.90E-21 |
| **40** | 4,9±1,1 | 0.085 | 468,59±627,4 | 7.70E-06 | 1,66±0,5 | 1.70E-29 | 2,64±0,9 | 0.0023 | 0,52±0,3 | 1.40E-34 | 9263,76±5736,3 | 8.20E-05 | 8,7±0,7 | 1.30E-07 | 4,12±0,4 | 0.039 | 139,51±2,9 | 0.012 |
| **41** | 4,91±1 | 0.046 | 125,02±259,3 | 2.20E-25 | 1,62±0,6 | 2.30E-14 | 2,65±0,9 | 0.00071 | 0,58±0,3 | 3.40E-13 | 9209,59±6040,6 | 0.0063 | 8,81±0,8 | 1.50E-18 | 4,14±0,4 | 0.19 | 139,48±2,8 | 0.024 |
| **42** | 5,53±1,4 | 1.60E-38 | 897,21±1350,2 | 0.31 | 1,56±0,6 | 0.00058 | 2,94±1,1 | 1.10E-14 | 0,82±0,4 | 3.60E-13 | 6876,76±4025,9 | 8.90E-09 | 7,9±1,1 | 5.60E-47 | 4,34±0,6 | 2.10E-17 | 139,11±3,3 | 0.79 |
| **43** | 4,97±1,1 | 0.0077 | 805,09±884,8 | 0.96 | 1,56±0,5 | 5.90E-06 | 2,59±0,9 | 0.063 | 0,73±0,4 | 0.73 | 9191,18±5591,3 | 0.0021 | 8,5±0,9 | 0.84 | 4,2±0,4 | 0.042 | 139,14±3 | 0.7 |
| **44** | 4,68±1 | 0.069 | 481,37±786,5 | 0.00041 | 1,46±0,4 | 0.13 | 2,65±0,9 | 0.011 | 0,54±0,3 | 1.50E-11 | 9379,55±5843,6 | 0.0014 | 8,85±0,8 | 3.40E-10 | 4,06±0,3 | 1.00E-04 | 139,34±2,8 | 0.26 |
| **45** | 4,72±1,2 | 0.026 | 1351,54±962,9 | 5.20E-15 | 1,1±0,3 | 6.40E-67 | 2,26±0,9 | 1.10E-06 | 1,12±0,5 | 8.00E-64 | 6759,86±3728 | 1.30E-10 | 8,38±1,1 | 0.04 | 4,28±0,5 | 2.80E-14 | 138,85±3,1 | 0.0062 |
| **46** | 4,84±1,1 | 0.8 | 638,16±711,1 | 0.13 | 1,64±0,6 | 1.20E-12 | 2,45±0,9 | 0.073 | 0,64±0,4 | 3.00E-05 | 7994,46±4395,7 | 0.71 | 8,2±0,9 | 2.80E-11 | 4,12±0,5 | 0.12 | 139,82±3,1 | 2.10E-08 |
| **47** | 4,95±1,4 | 0.7 | 919,26±937,7 | 0.39 | 1,36±0,6 | 8.90E-08 | 2,5±1 | 0.38 | 0,84±0,5 | 8.00E-07 | 7435,29±5780,9 | 1.20E-10 | 8,93±1,1 | 2.70E-18 | 4,13±0,4 | 0.18 | 138,42±3,4 | 2.30E-05 |
| **48** | 4,72±1,2 | 0.015 | 901,81±696,7 | 0.018 | 1,33±0,4 | 4.70E-06 | 2,49±1 | 0.12 | 0,75±0,4 | 0.027 | 8699,07±4586,4 | 0.0082 | 8,51±1 | 0.62 | 4,14±0,4 | 0.48 | 139,73±2,8 | 9.50E-05 |
| **49** | 4,81±1 | 0.98 | 111,74±205,7 | 1.10E-18 | 1,56±0,5 | 0.00019 | 2,54±0,8 | 0.62 | 0,61±0,3 | 5.60E-05 | 9892,85±6585,9 | 8.40E-05 | 8,69±0,9 | 2.00E-05 | 4,12±0,4 | 0.044 | 139,18±3 | 0.86 |
| **50** | 4,43±1,1 | 7.30E-08 | 854,85±575,3 | 0.0043 | 1,3±0,4 | 2.90E-07 | 2,24±0,9 | 3.30E-06 | 0,77±0,4 | 0.0027 | 9838,29±4867 | 1.50E-10 | 9,08±0,7 | 1.20E-23 | 4,11±0,4 | 0.17 | 140,08±2,5 | 1.30E-07 |
| **51** | 5,12±1 | 1.90E-08 | 722,2±674 | 0.77 | 1,53±0,5 | 0.00011 | 2,79±0,9 | 2.20E-08 | 0,74±0,4 | 0.13 | 8974,58±5807,4 | 0.052 | 8,33±0,8 | 0.00024 | 4,17±0,4 | 0.6 | 139,43±2,9 | 0.041 |
| **52** | 4,56±1,2 | 0.00014 | 786,41±534,8 | 0.14 | 1,27±0,4 | 5.10E-11 | 2,33±1 | 0.00021 | 0,78±0,4 | 0.0059 | 8859,46±4775,7 | 0.0048 | 8,68±0,9 | 0.00085 | 4,19±0,4 | 0.058 | 139,7±2,5 | 0.0097 |
| **53** | 4,59±1 | 4.00E-06 | 690,15±654,5 | 0.66 | 1,54±0,4 | 4.00E-08 | 2,42±0,8 | 0.088 | 0,56±0,3 | 7.40E-26 | 8623,22±5516,8 | 0.52 | 8,66±0,8 | 2.80E-05 | 4,13±0,4 | 0.16 | 139,65±2,8 | 4.70E-05 |
| **54** | 4,55±1,1 | 0.00021 | 891,12±726,6 | 0.13 | 1,32±0,4 | 0.00013 | 2,46±0,9 | 0.55 | 0,71±0,4 | 1 | 7976,73±5108,4 | 0.3 | 8,94±1 | 1.70E-11 | 4,11±0,4 | 0.29 | 139,34±2,9 | 0.42 |
| **55** | 5±1 | 0.0053 | 710,72±653,2 | 0.84 | 1,33±0,4 | 2.10E-05 | 2,83±0,9 | 8.50E-07 | 0,78±0,4 | 0.00023 | 8545,23±4953,7 | 0.23 | 8,67±1 | 0.0039 | 4,17±0,3 | 0.52 | 139,01±2,8 | 0.29 |
| **56** | 4,4±1,1 | 1.30E-13 | 793,52±597,6 | 0.28 | 1,29±0,4 | 9.00E-10 | 2,29±0,9 | 1.10E-06 | 0,7±0,5 | 0.09 | 8661,36±4230,8 | 0.0038 | 8,76±0,9 | 1.30E-07 | 4,19±0,5 | 0.26 | 139,52±2,9 | 0.024 |
| **57** | 4,76±1,2 | 0.23 | 471,69±571,7 | 0.0011 | 1,41±0,4 | 0.47 | 2,6±0,9 | 0.17 | 0,63±0,4 | 0.00097 | 9318,01±5639,6 | 0.00073 | 8,75±0,8 | 1.30E-07 | 4,1±0,4 | 0.014 | 139,97±2,9 | 1.60E-07 |
| **58** | 4,61±1 | 0.0074 | 523,27±628,9 | 0.0051 | 1,47±0,4 | 0.4 | 2,45±0,8 | 0.43 | 0,6±0,4 | 1.80E-06 | 9605,45±5758 | 0.0038 | 8,55±0,8 | 0.58 | 4,21±0,4 | 0.21 | 139,11±2,8 | 0.94 |
| **59** | 5,08±1,4 | 0.017 | 1085,36±989,2 | 0.041 | 1,6±0,5 | 5.00E-05 | 2,64±1,2 | 0.27 | 0,71±0,5 | 0.094 | 8335,86±5034,4 | 0.88 | 8,38±1 | 0.13 | 4,14±0,4 | 0.55 | 139,51±2,8 | 0.096 |
| **60** | 5,13±1,1 | 2.30E-06 | 665,38±689,7 | 0.49 | 1,67±0,5 | 1.70E-14 | 2,78±0,9 | 6.80E-05 | 0,59±0,3 | 1.80E-06 | 8232,62±5261,4 | 0.65 | 8,65±0,9 | 0.0039 | 4,17±0,4 | 0.84 | 139,42±3 | 0.23 |
| **61** | 4,85±1 | 0.61 | 1041±1173,9 | 0.16 | 1,49±0,5 | 0.14 | 2,62±0,9 | 0.17 | 0,68±0,4 | 1 | 7910,29±4540,1 | 0.33 | 8,29±1 | 0.00025 | 4,21±0,4 | 0.0067 | 139,76±2,9 | 0.00036 |
| **62** | 5,11±1,2 | 0.00014 | 955,41±678,8 | 0.0091 | 1,59±0,8 | 0.16 | 2,7±1 | 0.023 | 0,68±0,3 | 0.76 | 8494,77±5217,3 | 0.71 | 8,71±0,9 | 4.20E-05 | 4,18±0,4 | 0.69 | 138,57±3,1 | 8.00E-04 |
| **63** | 5,05±1,1 | 3.20E-05 | 761,57±837,6 | 0.89 | 1,38±0,4 | 0.0026 | 2,96±1,1 | 2.40E-18 | 0,67±0,3 | 0.58 | 9104,51±5593,2 | 0.0076 | 8,56±0,9 | 0.16 | 4,16±0,4 | 0.84 | 139,6±2,8 | 0.0065 |
| **64** | 4,37±1,1 | 3.70E-06 | 736,62±779 | 0.79 | 1,36±0,4 | 0.0093 | 2,21±0,9 | 2.40E-05 | 0,74±0,4 | 0.19 | 9301,51±5343 | 0.0011 | 8,77±0,9 | 0.00042 | 4,12±0,4 | 0.23 | 139,95±3,1 | 9.30E-05 |
| **65** | 5,23±1,3 | 1.00E-07 | 847,95±604,4 | 0.04 | 1,3±0,4 | 6.00E-09 | 2,95±1,1 | 1.50E-11 | 0,99±0,5 | 1.90E-29 | 9395,15±5119,7 | 8.00E-07 | 8,74±0,9 | 2.10E-05 | 4,1±0,4 | 0.038 | 139,48±2,7 | 0.09 |
| **66** | 4,78±1,2 | 0.42 | 624,21±732 | 0.051 | 1,5±0,6 | 0.64 | 2,38±0,9 | 0.011 | 0,69±0,4 | 0.27 | 7954,8±4543,8 | 0.36 | 8,46±1,1 | 0.86 | 4,21±0,5 | 0.12 | 139,3±3 | 0.43 |
| **67** | 4,91±1,3 | 0.78 | 481,49±557,2 | 0.0063 | 1,44±0,4 | 0.71 | 2,55±1 | 0.76 | 0,75±0,4 | 0.057 | 6826,12±4086,6 | 4.00E-10 | 8,39±0,8 | 0.063 | 4,14±0,4 | 0.55 | 139,64±2,8 | 0.0034 |
| **68** | 4,83±1,1 | 0.82 | 735,32±864,6 | 0.39 | 1,71±0,6 | 2.80E-16 | 2,38±0,9 | 0.0046 | 0,64±0,4 | 0.00013 | 7887,81±5524,5 | 0.03 | 8,16±0,9 | 8.50E-13 | 4,19±0,4 | 0.07 | 139,27±2,9 | 0.6 |
| **69** | 4,84±1,1 | 0.98 | 747,77±579 | 0.66 | 1,43±0,5 | 0.38 | 2,72±1 | 0.016 | 0,65±0,4 | 0.0084 | 9241,85±5393,9 | 0.00051 | 8,8±0,9 | 1.80E-05 | 4,06±0,4 | 0.00019 | 139,74±2,8 | 0.031 |
| **70** | 4,59±0,8 | 0.023 | 396,12±622,3 | 0.00017 | 1,54±0,4 | 0.006 | 2,49±0,7 | 0.99 | 0,51±0,3 | 1.80E-09 | 11971,17±6582 | 2.80E-17 | 8,93±0,7 | 6.40E-09 | 4,05±0,4 | 0.0033 | 139,69±2,3 | 0.1 |
| **71** | 4,81±1,2 | 0.76 | 856,69±799,5 | 0.51 | 1,68±0,6 | 2.90E-10 | 2,47±1 | 0.2 | 0,56±0,3 | 1.40E-09 | 7331,01±4970,4 | 0.00014 | 8,06±0,8 | 8.50E-13 | 4,1±0,4 | 0.022 | 139,21±3,5 | 0.45 |

**Supplementary Table 2 (cont.)**

| **Cluster** | **P/S Thyrotropin** | | **Weight** | | **Haemoglobin Beta A1C** | | **P/S Triglyceride** | | **P Creatinine** | | **BMI** | | **Height** | | **Diastolic blood pressure** | | **Systolic blood pressure** | |
| --- | --- | --- | --- | --- | --- | --- | --- | --- | --- | --- | --- | --- | --- | --- | --- | --- | --- | --- |
|  | **Mean±sd** | **P-value** | **Mean±sd** | **P-value** | **Mean±sd** | **P-value** | **Mean±sd** | **P-value** | **Mean±sd** | **P-value** | **Mean±sd** | **P-value** | **Mean±sd** | **P-value** | **Mean±sd** | **P-value** | **Mean±sd** | **P-value** |
| **1** | 1,9±2,1 | 0.00082 | 84,12±18,9 | 4.30E-168 | 8,5±1,4 | 6.30E-296 | 1,74±1,4 | 0.99 | 103,26±47,3 | 1.70E-110 | 26,95±5,6 | 0.0051 | 174,72±9,7 | 1.90E-37 | 77,06±10,8 | 1.20E-18 | 140,2±19,5 | 3.60E-110 |
| **2** | 1,76±1,6 | 0.45 | 77,29±16 | 3.00E-67 | 8,35±1,8 | 0.056 | 1,71±1,5 | 0.065 | 73,26±28,9 | 3.30E-72 | 24,91±5,2 | 1.70E-33 | 173,88±9,8 | 5.30E-05 | 77,84±9,9 | 0.66 | 129,97±16,9 | 2.20E-215 |
| **3** | 1,74±1,8 | 1.90E-08 | 87,27±20,3 | 5.00E-161 | 8,21±1,5 | 0.23 | 2,2±1,9 | 3.70E-40 | 106,73±43,9 | 1.20E-72 | 30,18±6,3 | 1.70E-33 | 171,84±10 | 0.39 | 77,15±11,8 | 4.60E-05 | 141,56±20,9 | 8.70E-56 |
| **4** | 1,79±1,8 | 0.013 | 77,64±17,9 | 7.00E-126 | 7,61±1,5 | 0 | 1,61±1,5 | 1.30E-16 | 72,2±31,6 | 1.40E-127 | 26,75±6,7 | 6.80E-06 | 167,64±10 | 2.90E-23 | 76,62±9,7 | 1.90E-31 | 125,62±16,9 | 0 |
| **5** | 1,91±2 | 0.96 | 87,67±19,7 | 1.40E-272 | 8,29±1,5 | 5.00E-06 | 1,9±1,5 | 3.30E-23 | 109,04±50,2 | 1.00E-118 | 29,01±5,8 | 6.00E-33 | 172,85±8,5 | 0.0046 | 78,49±10,3 | 2.30E-09 | 139,43±18,8 | 3.30E-26 |
| **6** | 2,03±2,2 | 0.94 | 83,44±15,3 | 1.80E-20 | 7,96±1,4 | 5.20E-21 | 2,22±2 | 6.10E-14 | 78,34±28 | 2.00E-06 | 28,37±5,1 | 0.00025 | 172,71±9,3 | 0.44 | 80,74±9,9 | 2.40E-35 | 141,42±18,4 | 2.30E-21 |
| **7** | 3,47±4 | 2.30E-47 | 78,36±16,9 | 1.20E-15 | 7,98±1,3 | 6.60E-25 | 1,62±1,2 | 0.00014 | 78,76±28 | 1.30E-16 | 26,36±6 | 0.0022 | 169,08±10,8 | 2.00E-09 | 78,19±10,1 | 0.037 | 135,08±18,6 | 7.70E-15 |
| **8** | 1,98±2,1 | 0.36 | 83,87±16,8 | 8.40E-42 | 8,35±1,4 | 2.90E-13 | 2,23±1,7 | 5.50E-37 | 88,3±34 | 0.15 | 29,3±5,1 | 7.40E-15 | 168,82±10 | 1.40E-07 | 75,2±10,7 | 5.70E-50 | 140,69±19,2 | 4.30E-32 |
| **9** | 1,79±1,9 | 3.30E-05 | 78,61±13,7 | 1.50E-10 | 7,85±1,1 | 5.50E-100 | 1,29±1 | 3.90E-64 | 77,38±18,9 | 5.10E-11 | 26±4 | 8.00E-04 | 173,42±8,6 | 0.043 | 83,37±10,4 | 0 | 151,9±19,3 | 0 |
| **10** | 1,79±1,2 | 0.36 | 76,94±15,7 | 5.00E-30 | 8±1,3 | 1.90E-19 | 1,43±0,9 | 4.00E-08 | 76,26±18,8 | 3.00E-09 | 24,24±4,8 | 1.40E-18 | 171,55±11,7 | 0.78 | 78,67±9,5 | 6.00E-05 | 132,86±17,4 | 1.50E-33 |
| **11** | 2,07±2,3 | 0.84 | 78,89±14,9 | 2.80E-08 | 7,76±1,3 | 3.10E-86 | 1,45±1 | 7.10E-09 | 78,73±22,5 | 3.00E-07 | 26,05±4 | 0.0083 | 174,88±8,6 | 2.50E-05 | 79,34±9,9 | 7.60E-16 | 134,27±16,7 | 1.30E-15 |
| **12** | 1,72±1,3 | 0.56 | 80,04±16,5 | 0.14 | 8,34±1,4 | 1.30E-12 | 1,55±1,2 | 0.00029 | 82,38±45,2 | 8.00E-16 | 27,32±6,1 | 0.69 | 169,07±10,3 | 1.30E-05 | 77,72±10,6 | 0.86 | 135,08±16,4 | 1.40E-08 |
| **13** | 1,95±2 | 0.93 | 77,92±14,8 | 1.70E-14 | 8,24±1,3 | 0.13 | 1,7±1,4 | 0.86 | 73,29±25,5 | 2.40E-33 | 27,92±5,3 | 0.079 | 169,01±10,8 | 2.60E-05 | 78,33±10,5 | 0.15 | 134,33±18,8 | 1.80E-16 |
| **14** | 1,92±2,2 | 0.6 | 81,13±16,9 | 0.00011 | 8,1±1,1 | 0.00041 | 1,34±0,9 | 2.40E-24 | 74,62±14,6 | 8.60E-14 | 24,96±4,4 | 4.30E-10 | 177,72±11 | 1.10E-12 | 79,02±8,8 | 1.80E-14 | 135,11±16,7 | 5.80E-12 |
| **15** | 2,16±2,7 | 0.86 | 78,48±16,6 | 7.00E-20 | 8,31±1,3 | 2.70E-08 | 1,48±1,2 | 4.30E-12 | 78,69±33,4 | 1.40E-20 | 27,14±4,6 | 0.69 | 171,76±9,1 | 0.47 | 77,86±9,6 | 0.59 | 134,59±17,5 | 1.70E-13 |
| **16** | 2,26±2,5 | 0.044 | 82,97±20,4 | 2.20E-07 | 8,24±1,5 | 0.88 | 1,93±1,5 | 2.30E-09 | 94,1±41,9 | 0.03 | 28,13±6,6 | 0.1 | 170,43±9,8 | 0.0084 | 78,09±10,9 | 0.11 | 137,72±19 | 0.44 |
| **17** | 2,25±1,9 | 7.30E-09 | 78,46±15 | 1.10E-10 | 8,57±1,4 | 4.60E-44 | 1,69±1,2 | 0.43 | 84,61±28,9 | 0.07 | 27,09±4,7 | 0.79 | 170,87±9,2 | 0.024 | 76,89±10,2 | 0.00024 | 138,07±18,7 | 0.017 |
| **18** | 2,08±2,2 | 0.96 | 79,08±16,9 | 2.60E-07 | 8,22±1,5 | 0.88 | 2±1,6 | 4.00E-11 | 114,87±51,9 | 2.50E-73 | 27,92±4,8 | 0.0047 | 170,54±8,8 | 0.012 | 76,54±10,5 | 1.80E-10 | 144,73±20,4 | 4.40E-100 |
| **19** | 2,08±1,8 | 0.042 | 76,37±15,2 | 1.90E-33 | 8,35±1,3 | 3.80E-09 | 1,49±1 | 9.70E-07 | 88,58±31 | 0.11 | 26,67±5,1 | 0.24 | 170,96±8,8 | 0.078 | 76,83±10,4 | 4.10E-05 | 141,63±19,8 | 2.40E-24 |
| **20** | 2±1,9 | 0.93 | 78,11±13,7 | 4.50E-10 | 8,33±1,4 | 0.00026 | 1,64±1,4 | 0.0081 | 88,09±35,4 | 0.68 | 27,89±4,6 | 0.028 | 168,98±10,8 | 0.00057 | 78,54±10,2 | 0.00039 | 142,85±18,7 | 8.70E-51 |
| **21** | 2,25±2,1 | 0.00097 | 93,43±23,3 | 2.60E-238 | 8,18±1,5 | 0.54 | 1,88±1,4 | 9.70E-07 | 107,63±41,6 | 2.60E-55 | 32,32±7,8 | 1.60E-31 | 173,53±10,4 | 0.019 | 76,39±10,8 | 1.20E-11 | 141,31±18,9 | 2.70E-33 |
| **22** | 1,71±1,6 | 0.0017 | 78,4±16,2 | 5.40E-06 | 8,47±1,3 | 1.10E-30 | 2,24±1,8 | 6.40E-25 | 91,39±40,6 | 0.28 | 26,76±5,7 | 0.64 | 168,11±10,4 | 9.20E-08 | 74,46±10,8 | 2.00E-51 | 143,55±20,6 | 9.20E-53 |
| **23** | 1,73±1,5 | 0.063 | 85,93±19,6 | 9.20E-51 | 8,02±1,4 | 7.00E-15 | 2,15±1,9 | 3.20E-13 | 104,3±58,9 | 2.50E-05 | 27,3±4,7 | 0.45 | 175,33±9,4 | 1.10E-07 | 77,85±10,3 | 0.86 | 137,06±17,9 | 0.59 |
| **24** | 2,58±3,3 | 2.30E-05 | 76,67±17,7 | 3.90E-46 | 8,37±1,3 | 2.20E-15 | 1,7±1,6 | 1.60E-05 | 89,83±37,1 | 0.53 | 25,43±4,6 | 8.00E-09 | 172,82±9,3 | 0.6 | 77,79±11 | 0.84 | 134,73±18,8 | 4.20E-10 |
| **25** | 1,63±1,5 | 7.30E-06 | 72,04±16,2 | 3.40E-164 | 8,45±1,6 | 3.00E-10 | 1,91±1,9 | 0.88 | 92,72±36,4 | 0.016 | 23,14±4,9 | 2.20E-19 | 172,04±9,9 | 0.73 | 78,36±10,1 | 0.0021 | 137,85±20,8 | 0.18 |
| **26** | 1,91±1,8 | 0.93 | 83,06±16,5 | 2.00E-27 | 8,12±1,3 | 0.027 | 1,74±1,1 | 0.0043 | 93,81±35 | 6.20E-06 | 28,9±4,7 | 2.60E-08 | 171,14±9,9 | 0.3 | 75,11±10,7 | 5.50E-40 | 143±20,1 | 4.00E-51 |
| **27** | 1,75±2 | 0.00017 | 77,27±16 | 5.10E-18 | 8,34±1,4 | 3.00E-08 | 1,94±1,5 | 4.10E-06 | 82,74±30,4 | 0.0064 | 27,8±6,1 | 0.58 | 169,4±8,5 | 1.00E-04 | 77,42±11,1 | 0.029 | 140,59±20,9 | 4.30E-11 |
| **28** | 1,49±0,9 | 0.042 | 81,46±19,6 | 0.8 | 8,13±1,5 | 0.00032 | 2,14±1,8 | 2.50E-07 | 73,65±21,3 | 4.50E-11 | 27,04±6,3 | 0.41 | 172,16±9,3 | 0.91 | 78,8±10,1 | 0.0037 | 133,02±16,8 | 1.40E-14 |
| **29** | 1,71±1,3 | 0.11 | 81,57±13,2 | 3.50E-10 | 7,92±1,2 | 1.20E-27 | 1,62±1,2 | 0.003 | 80,74±23,4 | 0.048 | 25,93±3,7 | 0.047 | 175,21±9,1 | 5.10E-05 | 77,97±9,4 | 0.21 | 136,79±17,6 | 0.25 |
| **30** | 2±1,7 | 0.027 | 74,83±25,3 | 2.70E-137 | 8,74±1,7 | 5.10E-52 | 1,75±1,4 | 0.58 | 77,93±39,8 | 1.20E-30 | 27,45±8,4 | 0.024 | 166,57±6,5 | 1.60E-16 | 78,14±10,6 | 0.74 | 125,28±17,8 | 2.80E-157 |
| **31** | 1,69±1,8 | 6.60E-07 | 76,66±16,1 | 2.00E-47 | 8,36±1,3 | 1.20E-15 | 1,44±0,9 | 2.80E-12 | 90,46±42,3 | 0.28 | 26,61±4,6 | 0.13 | 169,33±9 | 1.10E-05 | 77,49±9,1 | 0.37 | 135,35±16,7 | 1.50E-06 |
| **32** | 2,13±2,6 | 0.32 | 72,79±14,8 | 3.40E-147 | 8,4±1,4 | 2.90E-13 | 1,31±1 | 1.50E-26 | 75,2±28,9 | 8.60E-20 | 25,29±5,3 | 8.20E-09 | 170,81±9,2 | 0.1 | 75,77±10 | 7.80E-19 | 129,07±16,6 | 4.40E-100 |
| **33** | 1,86±2,2 | 0.063 | 84,67±18,7 | 2.80E-26 | 8,18±1,4 | 0.82 | 2,08±2 | 3.00E-04 | 83,06±29 | 0.24 | 29,21±6,3 | 0.00027 | 171,21±10,8 | 0.11 | 78,34±9,8 | 0.0066 | 137,29±17,2 | 0.56 |
| **34** | 1,88±1,8 | 0.3 | 102,72±22,6 | 0 | 8,13±1,4 | 0.012 | 1,86±1,2 | 0.00022 | 84,53±26,8 | 0.61 | 36,52±7,7 | 7.00E-33 | 173,99±8,4 | 0.12 | 80,27±9,8 | 3.20E-19 | 139,07±17,1 | 3.40E-05 |
| **35** | 1,74±1 | 0.099 | 90,8±17,7 | 3.90E-165 | 8,15±1,5 | 0.3 | 2,53±2,2 | 9.90E-38 | 119,42±49,9 | 2.50E-72 | 30,17±5,7 | 1.20E-14 | 172,27±10,2 | 0.78 | 79,03±11,2 | 4.50E-07 | 142,88±19,1 | 4.00E-37 |
| **36** | 2,07±1,9 | 0.16 | 76,49±15,3 | 5.30E-26 | 8,01±1,4 | 5.30E-11 | 1,64±1,1 | 0.81 | 69,84±28,9 | 4.60E-35 | 26,71±5,9 | 0.14 | 169,75±8,8 | 0.002 | 79,14±9,2 | 2.30E-06 | 129,03±17,2 | 1.40E-73 |
| **37** | 1,99±1,9 | 0.91 | 82,72±21,5 | 0.0031 | 8,2±1,6 | 0.5 | 2,12±1,9 | 9.60E-11 | 77,66±37,4 | 9.00E-15 | 28,74±6,5 | 0.0056 | 171,82±11,3 | 0.97 | 79,93±10 | 3.50E-16 | 137,92±20,4 | 0.26 |
| **38** | 1,41±0,8 | 0.0063 | 84,63±18,8 | 3.30E-19 | 8,25±1,5 | 0.74 | 2±1,4 | 1.70E-08 | 77,7±19 | 0.0018 | 27,22±5,8 | 0.26 | 174,22±12,3 | 0.01 | 79,34±9,1 | 3.90E-06 | 134,99±17,3 | 0.00065 |
| **39** | 1,84±1,8 | 0.2 | 74,46±19,9 | 9.20E-77 | 8,54±1,6 | 5.10E-23 | 1,95±1,4 | 2.80E-12 | 103,74±56,8 | 1.70E-06 | 24,62±4,9 | 8.30E-16 | 169,69±10,2 | 0.00052 | 78,17±10,3 | 0.041 | 140,43±20,1 | 7.40E-11 |
| **40** | 2,24±1,8 | 1.80E-07 | 83,66±16,1 | 5.90E-25 | 7,88±1,1 | 9.10E-28 | 1,15±0,7 | 1.00E-43 | 71,53±14,7 | 5.70E-21 | 27,48±5,6 | 0.96 | 173,68±11,1 | 0.23 | 77,86±9,2 | 0.39 | 136,6±16,6 | 0.23 |
| **41** | 1,68±1,1 | 0.63 | 78,23±16,2 | 1.10E-10 | 8,44±1,4 | 2.60E-17 | 1,32±0,8 | 2.10E-18 | 81,6±37,9 | 4.40E-07 | 24,53±3,5 | 1.40E-11 | 173,05±10,6 | 0.27 | 77,93±9,5 | 0.1 | 134,87±16,7 | 3.10E-09 |
| **42** | 1,51±1 | 0.002 | 74,32±15 | 3.70E-70 | 8,54±1,4 | 5.80E-32 | 2,09±1,6 | 3.90E-15 | 154,44±67,2 | 5.10E-73 | 25,3±4,4 | 1.70E-06 | 171,91±8,6 | 0.84 | 79,7±10,2 | 5.00E-15 | 142,9±19,9 | 4.80E-37 |
| **43** | 1,94±1,7 | 0.58 | 81,56±18,1 | 0.55 | 8,16±1,3 | 0.29 | 1,66±1,2 | 0.53 | 85,72±38,9 | 0.028 | 26,66±5 | 0.22 | 176,08±9,8 | 3.40E-05 | 78,3±9,9 | 0.024 | 137,27±17,2 | 0.6 |
| **44** | 1,77±1,8 | 0.82 | 73,71±12,3 | 5.60E-59 | 8,32±1,3 | 5.40E-05 | 1,25±0,9 | 2.00E-13 | 70,77±14,3 | 7.80E-11 | 24,56±3,7 | 3.60E-06 | 172,32±9,7 | 0.87 | 77,54±9,2 | 0.46 | 131,87±18 | 6.50E-27 |
| **45** | 1,73±1,5 | 0.01 | 96,32±19,9 | 3.30E-244 | 8,7±1,7 | 2.00E-31 | 3,01±2 | 3.00E-83 | 122,32±54,2 | 2.70E-44 | 32,32±5,4 | 1.50E-30 | 171,52±10 | 0.78 | 74,51±11,1 | 4.40E-35 | 134,05±20,9 | 5.60E-12 |
| **46** | 2,25±2,4 | 2.00E-05 | 77,85±15,6 | 2.80E-10 | 8,24±1,2 | 0.14 | 1,44±0,9 | 2.20E-07 | 96,41±42,9 | 0.0015 | 27,45±5,5 | 0.71 | 170,36±8,6 | 0.02 | 76,48±11 | 4.40E-07 | 141,88±22,1 | 1.10E-15 |
| **47** | 2,14±1,8 | 0.0042 | 88,72±19,9 | 4.10E-57 | 8,78±1,8 | 3.00E-40 | 2,69±2,7 | 4.70E-15 | 75,9±24,9 | 9.20E-11 | 29,39±8,1 | 0.074 | 174,15±9,5 | 0.1 | 79,4±10,7 | 1.70E-06 | 132,38±20 | 8.30E-27 |
| **48** | 2,4±2,2 | 0.00017 | 88,44±17,3 | 2.50E-71 | 8,06±1,3 | 0.00014 | 1,91±1,5 | 0.0069 | 92,74±35,5 | 0.00089 | 28,75±5,1 | 0.0012 | 174,93±8,7 | 0.0044 | 75,54±10,5 | 1.60E-13 | 136,71±17,6 | 0.68 |
| **49** | 2,03±1,9 | 0.56 | 75,51±12,2 | 5.30E-32 | 8,23±1,1 | 0.0054 | 1,63±1,8 | 5.60E-05 | 83,28±44,6 | 7.20E-07 | 23,43±3,9 | 2.00E-20 | 172,07±9,4 | 0.82 | 76,24±9,4 | 8.10E-09 | 131,91±16,4 | 2.30E-30 |
| **50** | 1,66±1,2 | 0.33 | 94,18±18,4 | 4.10E-89 | 7,86±1,6 | 4.30E-18 | 1,8±1,1 | 0.0041 | 82,84±22,5 | 0.87 | 29,93±4,8 | 2.10E-07 | 175,82±8 | 0.0015 | 80,26±9,9 | 6.90E-10 | 137,98±16,7 | 0.14 |
| **51** | 1,69±1,6 | 0.041 | 79,14±17,5 | 2.70E-06 | 8,32±1,2 | 6.00E-08 | 1,65±1,1 | 0.86 | 84,05±38,7 | 5.30E-06 | 27,62±5,9 | 0.69 | 172,54±9,3 | 0.97 | 78,05±10,1 | 0.36 | 136,13±17,8 | 0.027 |
| **52** | 1,69±1,3 | 0.57 | 85,38±17,1 | 2.40E-17 | 7,91±1,4 | 1.30E-12 | 1,8±1,4 | 0.055 | 80,9±22,6 | 0.23 | 26,59±4,8 | 0.24 | 175,67±9,4 | 0.00039 | 77,07±9,8 | 0.12 | 138,97±17,9 | 0.0084 |
| **53** | 2,13±2,4 | 0.65 | 77,72±12,6 | 8.90E-13 | 8,06±1 | 0.003 | 1,25±0,9 | 3.70E-33 | 94,29±38,2 | 0.00069 | 25,44±3,5 | 1.80E-06 | 174,51±8,1 | 0.00057 | 77,16±9,3 | 0.018 | 135,92±15,6 | 0.019 |
| **54** | 1,91±1,8 | 0.96 | 85,69±17,6 | 1.40E-22 | 8,05±1,4 | 0.0037 | 1,79±1,7 | 0.86 | 90,23±53 | 0.15 | 29,12±5,5 | 0.0031 | 173,52±10,8 | 0.29 | 80,39±9,4 | 3.50E-16 | 135,9±17,2 | 0.03 |
| **55** | 2,29±3,3 | 0.084 | 90,24±18,8 | 2.60E-70 | 8,66±1,7 | 1.10E-11 | 1,82±1,1 | 0.00054 | 72,22±17,7 | 2.70E-10 | 28,99±5,3 | 0.014 | 174,23±11,4 | 0.22 | 78,5±9,9 | 0.031 | 133,92±17,4 | 1.50E-08 |
| **56** | 1,59±1 | 0.34 | 83,25±11,7 | 5.40E-16 | 7,92±1,2 | 3.80E-11 | 1,84±1,9 | 0.21 | 102,72±27,9 | 5.70E-23 | 26,38±3,6 | 0.45 | 177,42±6,9 | 1.40E-07 | 77,05±10,5 | 0.047 | 142,88±19,1 | 1.70E-19 |
| **57** | 2,09±2,4 | 0.7 | 81,54±15,6 | 1.80E-05 | 8,55±1,3 | 1.30E-27 | 1,53±1,2 | 0.00064 | 81,4±24 | 0.089 | 26,06±4,2 | 0.12 | 172,52±9,1 | 0.85 | 79,95±9 | 6.50E-16 | 136,27±16 | 0.39 |
| **58** | 2,35±2,6 | 0.044 | 82,14±16,1 | 0.00082 | 7,71±1 | 1.20E-26 | 1,39±1,1 | 1.00E-08 | 77,96±17,8 | 0.015 | 26,47±4,8 | 0.58 | 174,73±11,8 | 0.077 | 80,01±9,3 | 3.50E-09 | 136,45±15,7 | 0.36 |
| **59** | 1,54±1 | 0.049 | 71,83±16,4 | 1.10E-69 | 8,48±1,4 | 2.00E-09 | 1,9±2 | 0.21 | 97,03±55,1 | 0.81 | 26,49±7,1 | 0.0081 | 166,68±7,5 | 6.80E-09 | 76,73±10,2 | 0.0021 | 136,36±20,7 | 0.21 |
| **60** | 2,74±3,2 | 0.0026 | 75,25±16,3 | 7.00E-38 | 8,4±1,2 | 4.00E-12 | 1,4±1 | 2.30E-07 | 82,48±26 | 0.39 | 27,06±5,8 | 0.65 | 171,09±11,7 | 0.57 | 76,94±10,6 | 0.012 | 140,26±20,9 | 1.00E-05 |
| **61** | 1,7±1,1 | 0.68 | 86,4±11,9 | 6.60E-52 | 7,9±1,3 | 1.80E-12 | 1,59±1 | 0.71 | 107,61±44,4 | 9.50E-16 | 26,67±3,7 | 0.81 | 178,02±6,8 | 1.20E-08 | 76,36±10,9 | 0.00016 | 141,32±19,3 | 1.60E-12 |
| **62** | 2,59±3,8 | 0.34 | 82,36±19,6 | 0.11 | 8,34±1,3 | 3.30E-05 | 1,72±1,6 | 0.64 | 78,82±25,9 | 0.00022 | 28,37±6,7 | 0.39 | 169,49±9,1 | 0.0054 | 79,5±9,8 | 5.90E-08 | 137,26±18,6 | 0.72 |
| **63** | 1,43±0,8 | 0.0053 | 82,61±16,8 | 7.80E-08 | 8,23±1,5 | 0.69 | 1,59±1,2 | 0.3 | 108,83±48,7 | 3.80E-14 | 26,82±5,1 | 0.39 | 175,32±7,2 | 0.00011 | 78,37±9,4 | 0.028 | 136,19±15,7 | 0.29 |
| **64** | 2,31±2 | 0.01 | 88,26±24 | 6.80E-13 | 7,98±1,5 | 1.90E-06 | 1,68±1 | 0.41 | 82,24±35,1 | 0.054 | 26,45±4 | 0.62 | 171,21±13,9 | 0.24 | 78,22±9,5 | 0.27 | 134,35±17,4 | 2.70E-05 |
| **65** | 1,5±0,9 | 0.044 | 82,18±15,7 | 5.10E-05 | 8,19±1,4 | 0.88 | 2,27±1,2 | 1.20E-27 | 89,37±37,4 | 0.53 | 28,22±4,9 | 0.1 | 170,11±9,4 | 0.1 | 77,31±10,8 | 0.26 | 134,4±18,1 | 6.50E-07 |
| **66** | 2,02±2,6 | 0.31 | 80,83±17,9 | 0.061 | 8,18±1,2 | 0.46 | 1,65±1,1 | 0.35 | 99,38±43,9 | 2.00E-06 | 26,28±4,4 | 0.1 | 174,9±10,7 | 0.018 | 76,9±10,8 | 0.0081 | 138,53±19,9 | 0.051 |
| **67** | 2,59±2,9 | 0.041 | 74,27±14,5 | 7.10E-51 | 8,38±1,2 | 9.20E-11 | 1,83±1,4 | 0.07 | 74,69±25,8 | 2.40E-16 | 26,49±5,3 | 0.091 | 166,29±8 | 2.10E-09 | 76,77±10,2 | 0.00063 | 134,52±19,9 | 7.80E-08 |
| **68** | 2,32±2 | 0.0014 | 71,91±15,6 | 9.30E-83 | 8,16±1,2 | 0.72 | 1,61±1,4 | 0.00013 | 93,54±35,5 | 0.0015 | 25,83±4,4 | 0.023 | 170,35±9,8 | 0.037 | 72,68±13,1 | 5.30E-51 | 131,8±23,6 | 1.70E-22 |
| **69** | 1,76±1,6 | 0.69 | 82,95±16,4 | 2.30E-06 | 8,01±1,4 | 4.60E-07 | 1,78±1,9 | 0.029 | 71,97±19,1 | 2.20E-10 | 27,68±6 | 0.7 | 172,07±8,8 | 0.78 | 81,07±11 | 1.80E-15 | 135,27±18,3 | 0.00038 |
| **70** | 1,49±0,6 | 0.61 | 79,35±14,5 | 0.14 | 8,26±1,3 | 0.14 | 1,21±0,9 | 1.20E-09 | 72,29±13,6 | 2.00E-05 | 24,03±3,8 | 3.50E-08 | 177,71±10,2 | 0.00011 | 75,77±10,4 | 4.20E-06 | 127,48±14,4 | 1.20E-38 |
| **71** | 1,92±2,2 | 0.45 | 70,5±14,3 | 1.40E-88 | 7,88±1,1 | 8.80E-14 | 1,29±0,9 | 5.50E-12 | 68,38±17,6 | 1.50E-21 | 26,45±5,1 | 0.39 | 164,82±7,3 | 5.50E-09 | 76,03±10,2 | 1.10E-07 | 137,02±19,5 | 0.73 |
