# Supplementary material for: Linking glycemic dysregulation in diabetes to symptoms, comorbidities, and genetics through EHR data mining
Source: eLife. 2019 Dec 10;8:e44941. doi: 10.7554/eLife.44941 (PMC6904221; doi:10.7554/eLife.44941)
Supplement: Supplementary file 3. [file elife-44941-supp3.docx]

**Supplementary Materials**

**Kirk and Simon et al.,**

**Linking glycemic dysregulation in diabetes to symptoms, comorbidities and genetics through EHR data mining.**

**Supplementary Table 3. Enrichment of drug prescriptions.** Drug prescriptions at ATC level 3 enriched in each of the 71 clusters with at least 50 individuals (hypergeometrical test with Benjamini-Hochberg corrected p-value <= 0.05) when accounting for sex and age distribution within the cluster.

| **Cluster** | **ATC Level** | **Counts in cluster** | **Frequency in cluster** | **P-value** | **BH adjusted p-value** |
| --- | --- | --- | --- | --- | --- |
| 1 | A02A | 69 | 7.040816327 | 1.71E-05 | 0.000610656 |
| 1 | A06A | 47 | 4.795918367 | 0.002642376 | 0.046315429 |
| 1 | A10A | 804 | 82.04081633 | 0.00087595 | 0.018516338 |
| 1 | A11D | 41 | 4.183673469 | 3.21E-05 | 0.001076941 |
| 1 | A11E | 107 | 10.91836735 | 6.45E-06 | 0.000256321 |
| 1 | A12B | 245 | 25 | 0.002048925 | 0.038291833 |
| 1 | B01A | 650 | 66.32653061 | 9.86E-06 | 0.0003685 |
| 1 | B03X | 39 | 3.979591837 | 3.54E-07 | 1.79E-05 |
| 1 | C02C | 22 | 2.244897959 | 0.000726505 | 0.015650351 |
| 1 | C03A | 330 | 33.67346939 | 0.001089339 | 0.022455227 |
| 1 | C03C | 345 | 35.20408163 | 2.34E-10 | 1.74E-08 |
| 1 | C09A | 534 | 54.48979592 | 4.49E-09 | 2.88E-07 |
| 1 | D06B | 19 | 1.93877551 | 8.21E-05 | 0.002464246 |
| 1 | D10A | 12 | 1.224489796 | 0.000148901 | 0.004001888 |
| 1 | J01C | 330 | 33.67346939 | 1.25E-51 | 7.05E-49 |
| 1 | J01F | 47 | 4.795918367 | 8.97E-10 | 6.17E-08 |
| 1 | J01M | 115 | 11.73469388 | 1.60E-44 | 6.46E-42 |
| 1 | N02A | 130 | 13.26530612 | 0.001996102 | 0.037678928 |
| 1 | P01A | 32 | 3.265306122 | 6.00E-12 | 5.05E-10 |
| 1 | P01B | 80 | 8.163265306 | 1.56E-05 | 0.000565389 |
| 1 | V03A | 29 | 2.959183673 | 7.94E-05 | 0.002408953 |
| 2 | A10A | 340 | 84.78802993 | 0.000418092 | 0.010127511 |
| 2 | A12B | 113 | 28.17955112 | 0.000566397 | 0.012786988 |
| 3 | A10B | 221 | 69.0625 | 8.74E-17 | 1.10E-14 |
| 3 | A12B | 155 | 48.4375 | 5.64E-28 | 1.38E-25 |
| 3 | B01A | 289 | 90.3125 | 5.96E-35 | 1.77E-32 |
| 3 | C01A | 174 | 54.375 | 1.10E-147 | 6.20E-144 |
| 3 | C01B | 21 | 6.5625 | 7.21E-17 | 9.25E-15 |
| 3 | C01D | 34 | 10.625 | 0.000291764 | 0.007286351 |
| 3 | C03A | 130 | 40.625 | 7.88E-06 | 0.000304652 |
| 3 | C03C | 199 | 62.1875 | 4.48E-42 | 1.58E-39 |
| 3 | C03D | 59 | 18.4375 | 1.33E-13 | 1.34E-11 |
| 3 | C03E | 15 | 4.6875 | 0.000409732 | 0.009967787 |
| 3 | C07A | 177 | 55.3125 | 1.08E-42 | 4.05E-40 |
| 3 | C08D | 65 | 20.3125 | 1.94E-23 | 3.53E-21 |
| 3 | C09A | 180 | 56.25 | 8.61E-05 | 0.00255618 |
| 3 | C09C | 133 | 41.5625 | 3.50E-07 | 1.78E-05 |
| 3 | C09D | 46 | 14.375 | 0.000117341 | 0.003311374 |
| 3 | C10A | 233 | 72.8125 | 0.000704323 | 0.015289226 |
| 3 | J01E | 27 | 8.4375 | 0.000128769 | 0.003562615 |
| 3 | M04A | 37 | 11.5625 | 8.63E-09 | 5.22E-07 |
| 3 | N02A | 55 | 17.1875 | 0.000136767 | 0.003747148 |
| 3 | N02B | 93 | 29.0625 | 3.12E-07 | 1.61E-05 |
| 3 | N05C | 40 | 12.5 | 1.02E-07 | 5.64E-06 |
| 4 | A03F | 29 | 9.477124183 | 9.88E-07 | 4.61E-05 |
| 4 | A10A | 260 | 84.96732026 | 0.001511467 | 0.030037741 |
| 4 | A11A | 36 | 11.76470588 | 4.83E-30 | 1.36E-27 |
| 4 | B03A | 67 | 21.89542484 | 8.44E-21 | 1.40E-18 |
| 4 | B03B | 106 | 34.64052288 | 9.11E-46 | 4.67E-43 |
| 4 | C02A | 24 | 7.843137255 | 0.000161363 | 0.004316268 |
| 4 | G03A | 31 | 10.13071895 | 6.52E-11 | 5.11E-09 |
| 4 | H04A | 52 | 16.99346405 | 6.76E-05 | 0.002108241 |
| 5 | A10B | 164 | 59.42028986 | 8.25E-06 | 0.000316685 |
| 5 | A12B | 92 | 33.33333333 | 1.90E-06 | 8.37E-05 |
| 5 | B01A | 224 | 81.15942029 | 1.83E-14 | 1.94E-12 |
| 5 | C02A | 33 | 11.95652174 | 3.89E-10 | 2.75E-08 |
| 5 | C02D | 7 | 2.536231884 | 0.000231333 | 0.005907881 |
| 5 | C03A | 132 | 47.82608696 | 4.09E-11 | 3.26E-09 |
| 5 | C03C | 156 | 56.52173913 | 1.82E-26 | 4.11E-24 |
| 5 | C07A | 90 | 32.60869565 | 3.88E-06 | 0.000167329 |
| 5 | C08C | 152 | 55.07246377 | 8.79E-18 | 1.24E-15 |
| 5 | C09A | 194 | 70.28985507 | 5.74E-17 | 7.53E-15 |
| 5 | C09B | 30 | 10.86956522 | 0.001933351 | 0.036649422 |
| 5 | C09C | 141 | 51.08695652 | 1.49E-15 | 1.75E-13 |
| 5 | C10A | 231 | 83.69565217 | 4.64E-13 | 4.29E-11 |
| 5 | D11A | 5 | 1.811594203 | 0.000530145 | 0.012163153 |
| 5 | G04B | 39 | 14.13043478 | 0.000162981 | 0.004338987 |
| 5 | M04A | 26 | 9.420289855 | 6.25E-05 | 0.001980239 |
| 6 | A10B | 162 | 69.82758621 | 2.93E-13 | 2.85E-11 |
| 6 | B01A | 165 | 71.12068966 | 0.000243981 | 0.006147439 |
| 7 | H03A | 78 | 34.66666667 | 1.14E-35 | 3.58E-33 |
| 8 | A10B | 148 | 66.07142857 | 1.81E-09 | 1.19E-07 |
| 8 | A12B | 71 | 31.69642857 | 0.000168211 | 0.004436962 |
| 8 | B01A | 183 | 81.69642857 | 1.52E-12 | 1.32E-10 |
| 8 | C01D | 99 | 44.19642857 | 2.04E-65 | 1.65E-62 |
| 8 | C03C | 88 | 39.28571429 | 2.05E-05 | 0.000724465 |
| 8 | C07A | 109 | 48.66071429 | 1.42E-20 | 2.23E-18 |
| 8 | C08D | 43 | 19.19642857 | 8.36E-15 | 9.25E-13 |
| 8 | C09A | 124 | 55.35714286 | 0.002300245 | 0.041643341 |
| 8 | C10A | 177 | 79.01785714 | 1.11E-06 | 5.14E-05 |
| 8 | N02B | 60 | 26.78571429 | 0.000460632 | 0.011003456 |
| 8 | P01B | 23 | 10.26785714 | 0.001232522 | 0.025022847 |
| 9 | A10A | 166 | 87.36842105 | 0.000737878 | 0.01583492 |
| 9 | C03A | 82 | 43.15789474 | 3.11E-05 | 0.001050925 |
| 9 | C08C | 89 | 46.84210526 | 1.49E-06 | 6.75E-05 |
| 9 | C09A | 107 | 56.31578947 | 0.00210975 | 0.039040754 |
| 9 | H04A | 34 | 17.89473684 | 0.000462052 | 0.011003456 |
| 11 | G03B | 6 | 3.571428571 | 2.09E-07 | 1.11E-05 |
| 12 | H02A | 15 | 9.615384615 | 0.000112207 | 0.003198458 |
| 12 | R01A | 15 | 9.615384615 | 3.06E-10 | 2.24E-08 |
| 12 | R03A | 98 | 62.82051282 | 1.06E-88 | 1.20E-85 |
| 12 | R03B | 63 | 40.38461538 | 6.08E-54 | 3.81E-51 |
| 12 | R03C | 38 | 24.35897436 | 1.03E-35 | 3.43E-33 |
| 12 | R03D | 21 | 13.46153846 | 3.65E-21 | 6.24E-19 |
| 12 | R06A | 16 | 10.25641026 | 0.000177344 | 0.004633938 |
| 14 | A10A | 131 | 90.34482759 | 8.35E-05 | 0.00249205 |
| 14 | H04A | 28 | 19.31034483 | 0.000409717 | 0.009967787 |
| 15 | D01A | 10 | 7.518796992 | 0.000655684 | 0.014399545 |
| 16 | A10B | 78 | 60.9375 | 0.000667347 | 0.014598868 |
| 16 | D05A | 6 | 4.6875 | 7.88E-09 | 4.83E-07 |
| 16 | L01B | 11 | 8.59375 | 1.80E-11 | 1.47E-09 |
| 16 | L04A | 11 | 8.59375 | 4.42E-05 | 0.001440716 |
| 17 | A02A | 14 | 11.11111111 | 0.000967848 | 0.020306817 |
| 17 | A03F | 18 | 14.28571429 | 3.65E-07 | 1.82E-05 |
| 17 | A06A | 26 | 20.63492063 | 1.46E-14 | 1.58E-12 |
| 17 | R06A | 14 | 11.11111111 | 0.000193858 | 0.005018956 |
| 18 | A02A | 13 | 10.48387097 | 0.002463925 | 0.043593716 |
| 18 | A02B | 36 | 29.03225806 | 4.59E-05 | 0.001490213 |
| 18 | A11C | 29 | 23.38709677 | 1.92E-05 | 0.000681749 |
| 18 | A11D | 9 | 7.258064516 | 0.001616491 | 0.03178911 |
| 18 | A12A | 22 | 17.74193548 | 0.001720291 | 0.033505211 |
| 18 | A12B | 50 | 40.32258065 | 1.15E-06 | 5.28E-05 |
| 18 | B01A | 95 | 76.61290323 | 6.63E-05 | 0.002079666 |
| 18 | B03A | 18 | 14.51612903 | 0.000548138 | 0.01242445 |
| 18 | C02A | 16 | 12.90322581 | 5.66E-06 | 0.000229928 |
| 18 | C03A | 58 | 46.77419355 | 2.84E-05 | 0.000969838 |
| 18 | C03C | 76 | 61.29032258 | 3.82E-16 | 4.59E-14 |
| 18 | C03D | 22 | 17.74193548 | 1.42E-05 | 0.000521795 |
| 18 | C03E | 8 | 6.451612903 | 0.001343589 | 0.026986535 |
| 18 | C07A | 44 | 35.48387097 | 0.00013959 | 0.003806014 |
| 18 | C08C | 68 | 54.83870968 | 1.44E-08 | 8.56E-07 |
| 18 | C09C | 59 | 47.58064516 | 5.66E-06 | 0.000229928 |
| 18 | C10A | 99 | 79.83870968 | 0.0001253 | 0.003483699 |
| 18 | N02A | 24 | 19.35483871 | 0.002134286 | 0.039365728 |
| 19 | B05B | 6 | 5 | 0.002688495 | 0.046832919 |
| 19 | S01B | 4 | 3.333333333 | 0.00216571 | 0.039815212 |
| 19 | S01E | 44 | 36.66666667 | 7.50E-45 | 3.26E-42 |
| 19 | S01F | 3 | 2.5 | 0.000840365 | 0.017830905 |
| 20 | C03A | 56 | 47.45762712 | 2.30E-05 | 0.000807533 |
| 20 | C08C | 53 | 44.91525424 | 0.00068246 | 0.014871831 |
| 20 | C09A | 78 | 66.10169492 | 6.16E-06 | 0.000246747 |
| 20 | C09C | 71 | 60.16949153 | 7.67E-13 | 6.98E-11 |
| 21 | A02B | 33 | 28.69565217 | 0.000120463 | 0.003382553 |
| 21 | A10B | 69 | 60 | 0.002362187 | 0.04224394 |
| 21 | A12B | 63 | 54.7826087 | 3.05E-15 | 3.51E-13 |
| 21 | B01A | 99 | 86.08695652 | 7.74E-10 | 5.39E-08 |
| 21 | C03A | 54 | 46.95652174 | 4.63E-05 | 0.001493823 |
| 21 | C03C | 74 | 64.34782609 | 1.85E-17 | 2.55E-15 |
| 21 | C03D | 17 | 14.7826087 | 0.001199034 | 0.024430868 |
| 21 | C07A | 39 | 33.91304348 | 0.000909051 | 0.019144339 |
| 21 | C08C | 57 | 49.56521739 | 1.45E-05 | 0.00052684 |
| 21 | C09A | 69 | 60 | 0.001450657 | 0.028931128 |
| 21 | C09C | 50 | 43.47826087 | 0.00046711 | 0.011077184 |
| 21 | C10A | 89 | 77.39130435 | 0.001786901 | 0.034303629 |
| 21 | D06B | 5 | 4.347826087 | 0.001760556 | 0.034029379 |
| 21 | G04C | 9 | 7.826086957 | 0.0020933 | 0.038992038 |
| 21 | J01C | 65 | 56.52173913 | 3.26E-24 | 6.35E-22 |
| 21 | J01F | 13 | 11.30434783 | 2.42E-07 | 1.27E-05 |
| 21 | J01M | 20 | 17.39130435 | 8.54E-11 | 6.61E-09 |
| 21 | M03B | 5 | 4.347826087 | 0.002588478 | 0.045512049 |
| 21 | M04A | 16 | 13.91304348 | 1.69E-05 | 0.000607542 |
| 21 | N02A | 26 | 22.60869565 | 0.000112799 | 0.003199184 |
| 21 | N02B | 46 | 40 | 1.54E-08 | 8.84E-07 |
| 21 | N06A | 33 | 28.69565217 | 0.000453021 | 0.010880225 |
| 21 | R05D | 11 | 9.565217391 | 0.000361857 | 0.008957551 |
| 22 | A10B | 71 | 62.28070175 | 0.000472269 | 0.011145696 |
| 22 | B01A | 93 | 81.57894737 | 5.90E-07 | 2.84E-05 |
| 22 | C01D | 20 | 17.54385965 | 5.44E-06 | 0.000225674 |
| 22 | C03C | 52 | 45.61403509 | 9.32E-06 | 0.000350636 |
| 22 | C09A | 70 | 61.40350877 | 0.000541461 | 0.012372503 |
| 22 | C10A | 91 | 79.8245614 | 0.000236402 | 0.006010156 |
| 23 | C08C | 53 | 48.18181818 | 7.60E-05 | 0.002330136 |
| 23 | D07A | 14 | 12.72727273 | 1.52E-12 | 1.32E-10 |
| 23 | D07B | 2 | 1.818181818 | 0.000473949 | 0.011145696 |
| 23 | J01C | 32 | 29.09090909 | 0.000218047 | 0.005593905 |
| 24 | A06A | 11 | 10.18518519 | 0.000636751 | 0.014069108 |
| 24 | A10A | 98 | 90.74074074 | 0.000447449 | 0.010792323 |
| 24 | A11C | 22 | 20.37037037 | 0.001415291 | 0.028325888 |
| 24 | A11D | 8 | 7.407407407 | 0.002565819 | 0.04525464 |
| 24 | N03A | 89 | 82.40740741 | 5.93E-78 | 5.58E-75 |
| 24 | N05B | 16 | 14.81481481 | 5.39E-06 | 0.000225161 |
| 24 | N06A | 33 | 30.55555556 | 0.000124376 | 0.003475139 |
| 25 | A09A | 44 | 41.50943396 | 3.72E-60 | 2.63E-57 |
| 25 | A10A | 102 | 96.22641509 | 1.41E-07 | 7.60E-06 |
| 25 | A11D | 16 | 15.09433962 | 1.06E-09 | 7.10E-08 |
| 25 | A11E | 22 | 20.75471698 | 5.76E-06 | 0.000232053 |
| 25 | N02A | 22 | 20.75471698 | 0.00126539 | 0.025598077 |
| 25 | N07B | 8 | 7.547169811 | 0.000499459 | 0.011648547 |
| 26 | A02A | 12 | 11.42857143 | 0.001721565 | 0.033505211 |
| 26 | A06A | 14 | 13.33333333 | 5.57E-06 | 0.000229639 |
| 26 | A12A | 19 | 18.0952381 | 0.002741464 | 0.047317496 |
| 26 | B01A | 77 | 73.33333333 | 0.00292245 | 0.049831751 |
| 26 | C01D | 14 | 13.33333333 | 0.002365182 | 0.04224394 |
| 26 | C03A | 51 | 48.57142857 | 2.40E-05 | 0.000836813 |
| 26 | C03C | 42 | 40 | 0.001898736 | 0.036327003 |
| 26 | C07A | 38 | 36.19047619 | 0.000245716 | 0.006163647 |
| 26 | C08C | 51 | 48.57142857 | 7.93E-05 | 0.002408953 |
| 26 | C09B | 17 | 16.19047619 | 0.000238399 | 0.006033737 |
| 26 | C10A | 93 | 88.57142857 | 1.53E-08 | 8.84E-07 |
| 26 | G03C | 9 | 8.571428571 | 0.000590511 | 0.013278262 |
| 26 | J01C | 28 | 26.66666667 | 0.002286442 | 0.041628004 |
| 26 | M01A | 25 | 23.80952381 | 9.19E-05 | 0.002674707 |
| 26 | N02A | 43 | 40.95238095 | 2.93E-16 | 3.60E-14 |
| 26 | N02B | 51 | 48.57142857 | 3.81E-13 | 3.59E-11 |
| 26 | N03A | 26 | 24.76190476 | 4.51E-07 | 2.21E-05 |
| 26 | N06A | 34 | 32.38095238 | 2.64E-05 | 0.000911174 |
| 26 | P01B | 13 | 12.38095238 | 0.002823839 | 0.048489617 |
| 27 | A02B | 55 | 52.88461538 | 1.50E-19 | 2.22E-17 |
| 27 | C03A | 45 | 43.26923077 | 0.001697084 | 0.033258131 |
| 27 | N02A | 21 | 20.19230769 | 0.00230944 | 0.041643712 |
| 29 | D01A | 10 | 9.803921569 | 7.43E-05 | 0.0022926 |
| 29 | D01B | 9 | 8.823529412 | 5.04E-09 | 3.20E-07 |
| 30 | D01A | 9 | 9 | 0.000327551 | 0.008144037 |
| 30 | G01A | 6 | 6 | 0.001003534 | 0.020977583 |
| 30 | G03A | 15 | 15 | 4.09E-08 | 2.31E-06 |
| 30 | N05A | 11 | 11 | 0.000607679 | 0.013610077 |
| 30 | N06A | 31 | 31 | 0.000147214 | 0.003975485 |
| 31 | A10A | 92 | 92 | 0.000183289 | 0.004767211 |
| 31 | A11E | 17 | 17 | 0.0008004 | 0.017111587 |
| 32 | A10A | 94 | 96.90721649 | 1.36E-07 | 7.37E-06 |
| 32 | G03A | 12 | 12.37113402 | 7.70E-06 | 0.000299782 |
| 32 | H04A | 32 | 32.98969072 | 3.60E-10 | 2.57E-08 |
| 33 | A02B | 26 | 27.08333333 | 0.001563669 | 0.03096613 |
| 33 | A10B | 61 | 63.54166667 | 0.000544942 | 0.012401816 |
| 33 | C10A | 79 | 82.29166667 | 8.80E-05 | 0.002599024 |
| 33 | M01A | 27 | 28.125 | 1.73E-06 | 7.75E-05 |
| 33 | N02A | 36 | 37.5 | 1.89E-12 | 1.62E-10 |
| 33 | N02B | 32 | 33.33333333 | 0.000168233 | 0.004436962 |
| 34 | A10B | 79 | 82.29166667 | 3.78E-13 | 3.59E-11 |
| 34 | B01A | 71 | 73.95833333 | 0.002915564 | 0.049831751 |
| 34 | C08C | 43 | 44.79166667 | 0.002243346 | 0.040975546 |
| 34 | C10A | 86 | 89.58333333 | 1.49E-08 | 8.78E-07 |
| 35 | A02B | 27 | 28.72340426 | 0.000476852 | 0.011167449 |
| 35 | A10B | 60 | 63.82978723 | 0.000507335 | 0.011735237 |
| 35 | A12B | 39 | 41.4893617 | 7.60E-06 | 0.000297782 |
| 35 | B01A | 74 | 78.72340426 | 8.84E-05 | 0.00259904 |
| 35 | C03C | 59 | 62.76595745 | 1.77E-13 | 1.75E-11 |
| 35 | C03D | 18 | 19.14893617 | 2.96E-05 | 0.001006857 |
| 35 | C07A | 45 | 47.87234043 | 5.74E-09 | 3.60E-07 |
| 35 | C08C | 42 | 44.68085106 | 0.002655562 | 0.046402446 |
| 35 | C09C | 50 | 53.19148936 | 4.66E-07 | 2.27E-05 |
| 35 | C10A | 83 | 88.29787234 | 1.29E-07 | 7.04E-06 |
| 35 | M01A | 23 | 24.46808511 | 0.000111549 | 0.003195861 |
| 35 | M04A | 50 | 53.19148936 | 3.98E-45 | 1.87E-42 |
| 36 | N02C | 12 | 12.90322581 | 5.64E-14 | 5.78E-12 |
| 37 | A10B | 63 | 70 | 5.03E-06 | 0.000213593 |
| 38 | A10B | 59 | 65.55555556 | 0.000196229 | 0.005057141 |
| 39 | A03F | 10 | 11.11111111 | 0.001178376 | 0.024184559 |
| 39 | A12A | 18 | 20 | 0.001090137 | 0.022455227 |
| 39 | N05C | 12 | 13.33333333 | 0.001935069 | 0.036649422 |
| 41 | A10A | 84 | 100 | 9.25E-10 | 6.29E-08 |
| 41 | H04A | 25 | 29.76190476 | 2.97E-07 | 1.55E-05 |
| 42 | A02A | 17 | 20.23809524 | 7.13E-08 | 3.98E-06 |
| 42 | A02B | 46 | 54.76190476 | 2.38E-17 | 3.19E-15 |
| 42 | A10A | 81 | 96.42857143 | 2.27E-06 | 9.92E-05 |
| 42 | A11C | 44 | 52.38095238 | 4.95E-22 | 8.73E-20 |
| 42 | A11E | 14 | 16.66666667 | 0.002733 | 0.047316102 |
| 42 | A12A | 30 | 35.71428571 | 1.66E-11 | 1.38E-09 |
| 42 | B03A | 17 | 20.23809524 | 1.09E-05 | 0.000403652 |
| 42 | B03X | 26 | 30.95238095 | 2.46E-26 | 5.35E-24 |
| 42 | B04A | 6 | 7.142857143 | 9.42E-05 | 0.002726167 |
| 42 | C02D | 5 | 5.952380952 | 3.80E-05 | 0.001248357 |
| 42 | C03C | 68 | 80.95238095 | 2.40E-25 | 4.84E-23 |
| 42 | C07A | 44 | 52.38095238 | 2.16E-10 | 1.63E-08 |
| 42 | C08C | 48 | 57.14285714 | 3.69E-07 | 1.83E-05 |
| 42 | C08D | 24 | 28.57142857 | 1.15E-12 | 1.03E-10 |
| 42 | D11A | 4 | 4.761904762 | 5.24E-05 | 0.001669966 |
| 42 | H02A | 74 | 88.0952381 | 3.73E-104 | 7.02E-101 |
| 42 | H05B | 3 | 3.571428571 | 4.73E-05 | 0.001518354 |
| 42 | J01C | 29 | 34.52380952 | 1.33E-05 | 0.000491782 |
| 42 | J05A | 11 | 13.0952381 | 2.49E-14 | 2.60E-12 |
| 42 | L04A | 74 | 88.0952381 | 2.80E-122 | 7.90E-119 |
| 42 | M04A | 13 | 15.47619048 | 3.34E-05 | 0.001103989 |
| 42 | N05C | 12 | 14.28571429 | 0.001051792 | 0.021905214 |
| 42 | P01B | 14 | 16.66666667 | 8.91E-05 | 0.002605694 |
| 42 | V03A | 6 | 7.142857143 | 0.001185394 | 0.024240454 |
| 45 | A02A | 10 | 12.5 | 0.002101055 | 0.039007749 |
| 45 | A02B | 22 | 27.5 | 0.002826556 | 0.048489617 |
| 45 | A10B | 52 | 65 | 0.000627817 | 0.013950389 |
| 45 | A12B | 54 | 67.5 | 6.69E-19 | 9.69E-17 |
| 45 | B01A | 64 | 80 | 0.000109717 | 0.003159405 |
| 45 | C01A | 31 | 38.75 | 8.19E-20 | 1.25E-17 |
| 45 | C01B | 6 | 7.5 | 9.23E-06 | 0.000349644 |
| 45 | C01D | 22 | 27.5 | 3.10E-10 | 2.24E-08 |
| 45 | C03C | 69 | 86.25 | 4.55E-29 | 1.17E-26 |
| 45 | C03D | 40 | 50 | 9.37E-27 | 2.20E-24 |
| 45 | C07A | 49 | 61.25 | 5.78E-15 | 6.53E-13 |
| 45 | C09A | 55 | 68.75 | 2.65E-05 | 0.000911174 |
| 45 | C10A | 64 | 80 | 0.001771472 | 0.034123499 |
| 45 | J01C | 23 | 28.75 | 0.001907932 | 0.03637962 |
| 45 | N02A | 23 | 28.75 | 4.48E-06 | 0.000191387 |
| 45 | N05C | 11 | 13.75 | 0.002302042 | 0.041643341 |
| 45 | R03A | 12 | 15 | 0.000821446 | 0.017495257 |
| 46 | C10A | 63 | 79.74683544 | 0.002228097 | 0.040829158 |
| 47 | N03A | 16 | 20.77922078 | 0.000638145 | 0.014069108 |
| 47 | N04A | 17 | 22.07792208 | 2.76E-26 | 5.78E-24 |
| 47 | N05A | 67 | 87.01298701 | 2.80E-90 | 3.95E-87 |
| 47 | N05B | 19 | 24.67532468 | 1.03E-10 | 7.82E-09 |
| 47 | N05C | 19 | 24.67532468 | 6.06E-09 | 3.76E-07 |
| 47 | N06A | 31 | 40.25974026 | 3.16E-07 | 1.62E-05 |
| 47 | N07B | 9 | 11.68831169 | 6.79E-06 | 0.000268066 |
| 48 | B01A | 63 | 81.81818182 | 3.34E-05 | 0.001103989 |
| 49 | A10A | 75 | 100 | 8.69E-09 | 5.22E-07 |
| 49 | G03A | 8 | 10.66666667 | 0.000724034 | 0.015650351 |
| 49 | H04A | 25 | 33.33333333 | 2.43E-08 | 1.39E-06 |
| 50 | A10B | 56 | 81.15942029 | 2.97E-09 | 1.93E-07 |
| 51 | L04A | 6 | 8.955223881 | 0.002042075 | 0.038290603 |
| 51 | M01A | 22 | 32.8358209 | 9.02E-07 | 4.28E-05 |
| 53 | A10A | 62 | 98.41269841 | 3.21E-06 | 0.000139555 |
| 53 | A11C | 21 | 33.33333333 | 6.76E-07 | 3.23E-05 |
| 53 | A12A | 19 | 30.15873016 | 1.79E-06 | 7.96E-05 |
| 54 | A02B | 21 | 34.42622951 | 0.000129764 | 0.003572616 |
| 56 | A10B | 41 | 68.33333333 | 0.000506736 | 0.011735237 |
| 56 | C10A | 49 | 81.66666667 | 0.002704392 | 0.046964889 |
| 56 | G04C | 24 | 40 | 1.16E-23 | 2.19E-21 |
| 59 | J01E | 11 | 19.64285714 | 8.46E-06 | 0.000322729 |
| 61 | B01A | 46 | 83.63636364 | 0.000140557 | 0.00381396 |
| 61 | C03C | 25 | 45.45454545 | 0.002021354 | 0.038028407 |
| 61 | G04C | 7 | 12.72727273 | 0.000373717 | 0.009170701 |
| 61 | L02A | 6 | 10.90909091 | 2.21E-11 | 1.78E-09 |
| 61 | L02B | 16 | 29.09090909 | 1.01E-20 | 1.63E-18 |
| 62 | A02B | 23 | 41.81818182 | 1.43E-06 | 6.52E-05 |
| 66 | A02A | 8 | 15.09433962 | 0.001728198 | 0.033518735 |
| 66 | A12B | 24 | 45.28301887 | 8.13E-05 | 0.002453941 |
| 66 | B01A | 42 | 79.24528302 | 0.002391863 | 0.042451808 |
| 66 | C01A | 11 | 20.75471698 | 7.08E-05 | 0.002195782 |
| 66 | C03C | 31 | 58.49056604 | 9.77E-07 | 4.60E-05 |
| 66 | C03D | 11 | 20.75471698 | 0.000509521 | 0.011737696 |
| 66 | C09A | 35 | 66.03773585 | 0.002316877 | 0.041644758 |
| 66 | C09C | 26 | 49.05660377 | 0.001323117 | 0.02667025 |
| 66 | H04A | 16 | 30.18867925 | 3.34E-05 | 0.001103989 |
| 66 | R05F | 2 | 3.773584906 | 0.001596896 | 0.031513579 |
| 67 | A03F | 7 | 13.20754717 | 0.002380485 | 0.042383147 |
| 67 | G03C | 7 | 13.20754717 | 0.000172446 | 0.004526907 |
| 67 | G03F | 5 | 9.433962264 | 0.001089553 | 0.022455227 |
| 67 | M01A | 18 | 33.96226415 | 5.14E-06 | 0.000216605 |
| 67 | N02A | 16 | 30.18867925 | 6.59E-05 | 0.002078838 |
| 67 | N02B | 29 | 54.71698113 | 1.34E-09 | 8.87E-08 |
| 67 | N06A | 19 | 35.8490566 | 0.000369156 | 0.009098318 |
| 67 | R06A | 8 | 15.09433962 | 0.000613406 | 0.01368404 |
| 71 | L02B | 20 | 40 | 4.38E-29 | 1.17E-26 |
