# Supplementary material for: Linking glycemic dysregulation in diabetes to symptoms, comorbidities, and genetics through EHR data mining
Source: eLife. 2019 Dec 10;8:e44941. doi: 10.7554/eLife.44941 (PMC6904221; doi:10.7554/eLife.44941)
Supplement: Supplementary file 4. [file elife-44941-supp4.docx]

**Supplementary Materials**

**Kirk and Simon et al.,**

**Linking glycemic dysregulation in diabetes to symptoms, comorbidities and genetics through EHR data mining.**

**Supplementary Table 4. Enrichment of ICD-10 and SDC-custom codes.** Diagnosis codes enriched in each of the 71 clusters with at least 50 individuals (binomial test, Benjamini-Hochberg corrected p-value <= 0.05) when accounting for sex and age distribution within the cluster.

| **Cluster** | **ICD10 or SDC term** | **Observed** | **Expected** | **Adj. P-value** |  | **Cluster** | **ICD10 or SDC term** | **Observed** | **Expected** | **Adj. P-value** |
| --- | --- | --- | --- | --- | --- | --- | --- | --- | --- | --- |
| 1 | A46 | 54 | 1651 | 1.89E-04 |  | 24 | I64 | 9 | 140 | 6.05E-03 |
| 1 | E10 | 506 | 120137 | 9.32E-51 |  | 24 | R42 | 40 | 8312 | 4.73E-02 |
| 1 | E66 | 165 | 17404 | 1.38E-10 |  | 24 | R45 | 49 | 12146 | 2.40E-02 |
| 1 | E87 | 127 | 10530 | 2.80E-02 |  | 24 | R47 | 5 | 26 | 2.74E-02 |
| 1 | F98 | 61 | 2495 | 3.96E-02 |  | 24 | R73 | 84 | 39796 | 2.34E-02 |
| 1 | H36 | 197 | 18993 | 2.84E-16 |  | 24 | sdcC06 | 44 | 7263 | 8.64E-07 |
| 1 | I10 | 449 | 199537 | 4.07E-02 |  | 24 | sdcC16 | 13 | 780 | 6.04E-03 |
| 1 | I83 | 90 | 1924 | 2.67E-23 |  | 24 | sdcC26 | 20 | 1134 | 5.40E-05 |
| 1 | I89 | 5 | 5 | 2.51E-02 |  | 24 | sdcK05 | 27 | 3255 | 3.26E-02 |
| 1 | J34 | 9 | 19 | 2.19E-04 |  | 24 | sdcL02 | 20 | 833 | 1.39E-03 |
| 1 | L03 | 8 | 20 | 2.41E-02 |  | 24 | sdcM031 | 89 | 42942 | 5.69E-03 |
| 1 | L89 | 207 | 7268 | 1.35E-89 |  | 24 | sdcM101 | 82 | 38138 | 2.92E-02 |
| 1 | L97 | 385 | 37735 | 1.19E-132 |  | 24 | sdcM132 | 12 | 287 | 2.58E-02 |
| 1 | M14 | 38 | 1051 | 2.63E-05 |  | 24 | sdcN05 | 7 | 93 | 1.05E-02 |
| 1 | M20 | 29 | 238 | 5.88E-09 |  | 24 | sdcN11 | 53 | 16493 | 3.28E-02 |
| 1 | O92 | 21 | 195 | 1.26E-02 |  | 24 | sdcO03 | 8 | 83 | 1.15E-03 |
| 1 | R23 | 411 | 59527 | 4.21E-81 |  | 25 | E13 | 93 | 1739 | 1.80E-142 |
| 1 | R80 | 257 | 51842 | 4.04E-03 |  | 25 | E14 | 44 | 7855 | 5.36E-06 |
| 1 | Z89 | 163 | 5302 | 1.41E-68 |  | 25 | K80 | 8 | 78 | 7.50E-03 |
| 1 | sdcC17 | 152 | 10566 | 1.12E-17 |  | 25 | K85 | 4 | 8 | 1.42E-03 |
| 1 | sdcC28 | 206 | 33321 | 2.62E-02 |  | 25 | K86 | 84 | 1459 | 2.75E-125 |
| 1 | sdcC33 | 125 | 11508 | 1.77E-04 |  | 25 | sdcM021 | 73 | 29038 | 3.23E-05 |
| 1 | sdcE01 | 466 | 172423 | 4.47E-03 |  | 25 | sdcM151 | 58 | 17968 | 1.19E-03 |
| 1 | sdcE02 | 25 | 226 | 4.13E-03 |  | 26 | E55 | 7 | 112 | 3.53E-02 |
| 1 | sdcE04 | 264 | 44306 | 4.09E-12 |  | 26 | F40 | 2 | 6 | 4.91E-02 |
| 1 | sdcE05 | 116 | 7868 | 9.15E-03 |  | 26 | M47 | 5 | 29 | 1.21E-02 |
| 1 | sdcE07 | 293 | 41749 | 6.95E-24 |  | 26 | M48 | 104 | 2382 | 1.98E-146 |
| 1 | sdcE08 | 241 | 25523 | 9.09E-27 |  | 26 | M51 | 4 | 17 | 3.78E-03 |
| 1 | sdcE09 | 565 | 210505 | 5.12E-25 |  | 26 | M54 | 44 | 3157 | 1.65E-16 |
| 1 | sdcE12 | 65 | 1976 | 4.61E-11 |  | 26 | Q78 | 2 | 2 | 1.27E-02 |
| 1 | sdcE13 | 16 | 74 | 4.41E-02 |  | 26 | R52 | 91 | 39221 | 1.44E-09 |
| 1 | sdcF02 | 367 | 42875 | 1.39E-93 |  | 26 | sdcN11 | 75 | 29438 | 1.94E-04 |
| 1 | sdcF03 | 57 | 612 | 3.83E-23 |  | 26 | sdcN12 | 62 | 18086 | 2.03E-03 |
| 1 | sdcF04 | 379 | 50371 | 4.82E-79 |  | 27 | K06 | 4 | 10 | 8.33E-05 |
| 1 | sdcF05 | 394 | 83879 | 3.03E-28 |  | 27 | K25 | 92 | 3143 | 1.31E-111 |
| 1 | sdcF12 | 26 | 362 | 2.97E-02 |  | 27 | K30 | 13 | 592 | 1.08E-02 |
| 1 | sdcF13 | 6 | 10 | 4.01E-02 |  | 27 | K92 | 6 | 15 | 1.96E-04 |
| 1 | sdcF18 | 216 | 17019 | 4.76E-37 |  | 27 | R11 | 43 | 8251 | 2.58E-03 |
| 1 | sdcF19 | 274 | 31795 | 5.76E-41 |  | 27 | Z74 | 7 | 59 | 4.86E-04 |
| 1 | sdcF21 | 215 | 12038 | 2.19E-64 |  | 28 | H53 | 104 | 9790 | 2.98E-93 |
| 1 | sdcF22 | 40 | 502 | 1.28E-09 |  | 29 | B35 | 99 | 3677 | 1.78E-134 |
| 1 | sdcK02 | 218 | 29886 | 1.89E-09 |  | 29 | sdcM021 | 67 | 28979 | 1.73E-03 |
| 1 | sdcK03 | 423 | 137648 | 1.99E-06 |  | 29 | sdcM031 | 82 | 49785 | 1.45E-02 |
| 1 | sdcK05 | 268 | 47267 | 1.48E-14 |  | 30 | E66 | 19 | 1674 | 5.99E-03 |
| 1 | sdcK06 | 137 | 8401 | 2.07E-11 |  | 30 | F32 | 11 | 84 | 6.61E-06 |
| 1 | sdcK07 | 80 | 4305 | 3.00E-04 |  | 30 | F50 | 100 | 3400 | 1.37E-116 |
| 1 | sdcK08 | 427 | 115465 | 7.61E-16 |  | 30 | R40 | 4 | 13 | 2.10E-02 |
| 1 | sdcK12 | 59 | 1525 | 2.17E-07 |  | 30 | R63 | 13 | 284 | 5.22E-03 |
| 1 | sdcM021 | 530 | 198631 | 1.92E-06 |  | 30 | T73 | 47 | 8412 | 3.24E-03 |
| 1 | sdcM151 | 448 | 126898 | 7.03E-09 |  | 30 | sdcL03 | 65 | 21162 | 8.93E-03 |
| 1 | sdcN08 | 251 | 55148 | 2.26E-03 |  | 31 | E10 | 50 | 14982 | 3.23E-03 |
| 1 | sdcN10 | 215 | 23834 | 4.48E-24 |  | 31 | G56 | 100 | 3880 | 1.25E-133 |
| 1 | sdcN11 | 530 | 218639 | 5.47E-13 |  | 31 | G63 | 5 | 40 | 4.40E-02 |
| 1 | sdcN12 | 396 | 128472 | 1.45E-06 |  | 31 | H36 | 22 | 2015 | 2.04E-02 |
| 1 | sdcN13 | 320 | 62077 | 1.47E-28 |  | 31 | M65 | 20 | 646 | 2.08E-08 |
| 1 | sdcO04 | 151 | 14431 | 9.96E-07 |  | 31 | R52 | 76 | 37115 | 1.75E-05 |
| 2 | E14 | 129 | 21821 | 1.20E-06 |  | 31 | R80 | 32 | 6432 | 4.30E-02 |
| 2 | E86 | 23 | 439 | 1.86E-02 |  | 31 | sdcC19 | 19 | 1385 | 2.57E-02 |
| 2 | E87 | 52 | 3164 | 4.94E-03 |  | 31 | sdcE09 | 63 | 23802 | 5.80E-04 |
| 2 | R35 | 281 | 24158 | 5.72E-158 |  | 31 | sdcF15 | 58 | 21248 | 3.56E-03 |
| 2 | R63 | 141 | 4175 | 1.66E-85 |  | 31 | sdcK03 | 48 | 14147 | 1.82E-02 |
| 2 | R73 | 294 | 136212 | 3.20E-04 |  | 31 | sdcM021 | 74 | 31746 | 4.90E-06 |
| 2 | R82 | 199 | 11494 | 5.78E-97 |  | 31 | sdcM031 | 87 | 52578 | 9.65E-05 |
| 2 | T73 | 222 | 47039 | 2.27E-29 |  | 31 | sdcM101 | 79 | 43750 | 1.48E-02 |
| 2 | sdcM042 | 105 | 8997 | 1.12E-08 |  | 31 | sdcM151 | 59 | 19093 | 3.07E-04 |
| 2 | sdcM08 | 49 | 1026 | 1.01E-13 |  | 31 | sdcN04 | 11 | 267 | 7.37E-04 |
| 2 | sdcM101 | 277 | 125087 | 1.12E-02 |  | 31 | sdcN10 | 20 | 1669 | 1.18E-02 |
| 2 | sdcO090 | 157 | 32909 | 3.59E-03 |  | 31 | sdcN11 | 60 | 23500 | 2.82E-03 |
| 2 | sdcO15 | 87 | 4858 | 1.19E-20 |  | 31 | sdcN12 | 53 | 16020 | 6.35E-05 |
| 2 | sdcS5 | 10 | 118 | 4.95E-02 |  | 32 | E10 | 49 | 12294 | 9.90E-04 |
| 3 | E11 | 233 | 92138 | 9.05E-10 |  | 32 | E61 | 6 | 63 | 2.89E-02 |
| 3 | E66 | 57 | 6092 | 3.20E-03 |  | 32 | E73 | 12 | 48 | 1.22E-10 |
| 3 | I35 | 51 | 847 | 5.96E-25 |  | 32 | G56 | 10 | 457 | 2.45E-02 |
| 3 | I48 | 266 | 16305 | 3.75E-210 |  | 32 | K90 | 93 | 1968 | 5.99E-123 |
| 3 | I49 | 16 | 185 | 8.18E-03 |  | 32 | R52 | 57 | 20510 | 1.80E-02 |
| 3 | I50 | 49 | 2001 | 2.40E-08 |  | 32 | R73 | 78 | 36628 | 3.22E-02 |
| 3 | M80 | 3 | 2 | 2.85E-02 |  | 32 | sdcE07 | 23 | 3054 | 4.85E-02 |
| 3 | R06 | 58 | 5420 | 3.85E-02 |  | 32 | sdcM021 | 62 | 21951 | 8.23E-03 |
| 3 | R70 | 25 | 220 | 5.50E-09 |  | 32 | sdcM031 | 79 | 38673 | 4.83E-02 |
| 3 | sdcC17 | 55 | 3726 | 9.19E-03 |  | 32 | sdcM101 | 78 | 34683 | 2.55E-03 |
| 3 | sdcC31 | 42 | 2390 | 1.74E-02 |  | 32 | sdcM151 | 54 | 15260 | 5.16E-03 |
| 3 | sdcK02 | 75 | 10028 | 4.18E-02 |  | 32 | sdcN07 | 26 | 1662 | 4.12E-06 |
| 3 | sdcK03 | 177 | 54729 | 2.01E-04 |  | 32 | sdcN11 | 48 | 15060 | 3.93E-04 |
| 3 | sdcK05 | 88 | 13317 | 2.60E-02 |  | 32 | sdcN15 | 47 | 11527 | 3.70E-02 |
| 3 | sdcK08 | 164 | 43000 | 1.09E-03 |  | 32 | sdcO16 | 34 | 5554 | 3.40E-02 |
| 3 | sdcM18 | 8 | 197 | 4.54E-02 |  | 33 | M54 | 96 | 7141 | 8.14E-102 |
| 4 | O03 | 48 | 1490 | 1.10E-15 |  | 33 | R52 | 64 | 31637 | 4.15E-02 |
| 4 | O24 | 134 | 16252 | 4.17E-35 |  | 34 | E66 | 26 | 3211 | 2.70E-04 |
| 4 | R11 | 256 | 53695 | 4.52E-73 |  | 34 | E78 | 59 | 32638 | 1.97E-02 |
| 4 | R45 | 142 | 36771 | 9.02E-03 |  | 34 | F33 | 4 | 27 | 3.82E-02 |
| 4 | Z33 | 27 | 389 | 1.24E-10 |  | 34 | G47 | 96 | 4819 | 1.66E-134 |
| 4 | sdcK08 | 173 | 39054 | 1.76E-14 |  | 34 | I10 | 60 | 34006 | 7.61E-03 |
| 4 | sdcN07 | 50 | 3000 | 1.90E-04 |  | 34 | R06 | 19 | 2286 | 2.82E-02 |
| 4 | sdcO04 | 65 | 5364 | 8.60E-04 |  | 34 | sdcL12 | 19 | 1714 | 1.25E-02 |
| 4 | sdcO100 | 28 | 858 | 2.86E-03 |  | 34 | sdcM18 | 9 | 178 | 9.63E-04 |
| 5 | E11 | 170 | 83386 | 3.85E-13 |  | 35 | M10 | 94 | 3476 | 1.94E-131 |
| 5 | E66 | 46 | 5393 | 1.56E-02 |  | 35 | R52 | 67 | 34795 | 3.32E-03 |
| 5 | E87 | 48 | 5372 | 2.27E-03 |  | 35 | R80 | 36 | 9271 | 1.62E-02 |
| 5 | H36 | 72 | 7986 | 6.14E-11 |  | 36 | G43 | 93 | 2961 | 6.05E-128 |
| 5 | I10 | 140 | 72174 | 4.52E-02 |  | 36 | R51 | 42 | 7929 | 1.44E-04 |
| 5 | I95 | 53 | 6572 | 7.78E-04 |  | 37 | E11 | 48 | 15283 | 1.60E-05 |
| 5 | R42 | 101 | 28550 | 2.83E-03 |  | 37 | E14 | 33 | 5210 | 6.09E-03 |
| 5 | R53 | 40 | 4124 | 1.79E-02 |  | 37 | R35 | 23 | 1789 | 1.34E-03 |
| 5 | R80 | 274 | 63180 | 1.49E-172 |  | 37 | R43 | 2 | 3 | 3.53E-02 |
| 5 | sdcC12 | 160 | 79526 | 1.30E-04 |  | 37 | R81 | 89 | 1677 | 3.01E-138 |
| 5 | sdcC14 | 192 | 112385 | 1.44E-08 |  | 37 | R82 | 33 | 2214 | 1.84E-12 |
| 5 | sdcC28 | 109 | 20179 | 2.18E-16 |  | 37 | sdcL03 | 60 | 26254 | 2.18E-02 |
| 5 | sdcC35 | 54 | 4470 | 1.83E-11 |  | 37 | sdcL12 | 16 | 913 | 1.80E-02 |
| 5 | sdcC36 | 46 | 4759 | 2.00E-06 |  | 37 | sdcO15 | 18 | 938 | 1.39E-04 |
| 5 | sdcE04 | 81 | 16603 | 1.39E-04 |  | 38 | H53 | 21 | 2137 | 2.25E-02 |
| 5 | sdcE05 | 37 | 3146 | 3.51E-02 |  | 38 | R19 | 90 | 7132 | 4.27E-96 |
| 5 | sdcE07 | 79 | 13155 | 3.17E-05 |  | 39 | D72 | 41 | 848 | 2.62E-37 |
| 5 | sdcE08 | 73 | 8889 | 7.88E-09 |  | 39 | E10 | 42 | 8311 | 7.33E-03 |
| 5 | sdcE09 | 189 | 81280 | 1.27E-17 |  | 39 | E86 | 59 | 1115 | 2.46E-63 |
| 5 | sdcE13 | 8 | 54 | 2.60E-02 |  | 39 | E87 | 23 | 1691 | 1.68E-04 |
| 5 | sdcF18 | 50 | 4713 | 2.25E-05 |  | 39 | R11 | 43 | 6815 | 1.56E-05 |
| 5 | sdcK02 | 194 | 31576 | 8.00E-93 |  | 39 | R23 | 24 | 3032 | 3.99E-02 |
| 5 | sdcK03 | 171 | 66507 | 1.59E-17 |  | 39 | R40 | 4 | 8 | 9.93E-03 |
| 5 | sdcK05 | 196 | 39121 | 2.96E-82 |  | 39 | R53 | 19 | 1215 | 7.21E-03 |
| 5 | sdcK06 | 83 | 6400 | 1.63E-27 |  | 39 | R82 | 20 | 776 | 4.22E-04 |
| 5 | sdcK07 | 61 | 3446 | 3.15E-19 |  | 39 | sdcF05 | 42 | 7722 | 6.52E-05 |
| 5 | sdcK08 | 171 | 53287 | 1.45E-23 |  | 39 | sdcM042 | 28 | 1358 | 8.41E-06 |
| 5 | sdcK11 | 30 | 1290 | 1.52E-05 |  | 39 | sdcN05 | 7 | 74 | 4.31E-02 |
| 5 | sdcK12 | 20 | 395 | 2.32E-03 |  | 40 | L01 | 2 | 3 | 2.93E-02 |
| 5 | sdcL02 | 32 | 1763 | 5.89E-03 |  | 40 | M72 | 32 | 531 | 3.72E-35 |
| 5 | sdcL05 | 184 | 104846 | 9.15E-03 |  | 40 | M77 | 68 | 1422 | 5.76E-92 |
| 5 | sdcL06 | 79 | 17779 | 1.92E-02 |  | 40 | R52 | 65 | 34396 | 1.20E-03 |
| 5 | sdcL12 | 42 | 3981 | 1.13E-03 |  | 41 | E10 | 61 | 17051 | 5.80E-14 |
| 5 | sdcM19 | 15 | 349 | 3.71E-03 |  | 41 | E15 | 19 | 452 | 2.79E-10 |
| 5 | sdcN11 | 147 | 73254 | 9.71E-03 |  | 41 | R40 | 4 | 21 | 6.33E-03 |
| 5 | sdcN12 | 115 | 44723 | 3.81E-02 |  | 41 | R45 | 42 | 10096 | 1.02E-03 |
| 5 | sdcN13 | 79 | 18827 | 4.64E-05 |  | 41 | R73 | 72 | 40995 | 1.00E-04 |
| 5 | sdcN15 | 107 | 35047 | 3.26E-02 |  | 41 | T38 | 84 | 2247 | 3.96E-126 |
| 5 | sdcO16 | 98 | 22948 | 2.99E-05 |  | 41 | sdcE01 | 52 | 19992 | 7.34E-04 |
| 6 | E11 | 158 | 65781 | 8.42E-17 |  | 41 | sdcE04 | 25 | 4496 | 3.26E-02 |
| 6 | E78 | 139 | 62008 | 7.14E-05 |  | 41 | sdcE09 | 57 | 22148 | 2.22E-06 |
| 6 | I10 | 131 | 60341 | 3.96E-03 |  | 41 | sdcE11 | 16 | 1279 | 2.80E-02 |
| 7 | E03 | 77 | 3282 | 2.85E-40 |  | 41 | sdcM021 | 63 | 26184 | 1.89E-06 |
| 7 | F99 | 3 | 5 | 3.72E-02 |  | 41 | sdcM031 | 79 | 45248 | 4.63E-08 |
| 7 | R00 | 107 | 10529 | 6.29E-41 |  | 41 | sdcM101 | 73 | 40340 | 2.36E-05 |
| 7 | R53 | 79 | 5884 | 5.14E-25 |  | 41 | sdcM132 | 84 | 2849 | 8.90E-116 |
| 8 | E11 | 155 | 61690 | 5.04E-09 |  | 41 | sdcM151 | 72 | 23518 | 2.68E-18 |
| 8 | E78 | 130 | 53702 | 6.55E-03 |  | 41 | sdcN15 | 42 | 14335 | 2.68E-02 |
| 8 | I20 | 161 | 11753 | 1.33E-109 |  | 41 | sdcO03 | 10 | 83 | 9.96E-07 |
| 8 | I21 | 14 | 309 | 6.72E-03 |  | 42 | E10 | 62 | 16756 | 1.84E-12 |
| 8 | I25 | 138 | 11790 | 1.15E-79 |  | 42 | E87 | 20 | 1512 | 4.29E-04 |
| 8 | N39 | 13 | 141 | 2.87E-06 |  | 42 | H36 | 27 | 2643 | 2.36E-06 |
| 8 | R52 | 144 | 64566 | 4.34E-02 |  | 42 | I83 | 10 | 164 | 6.27E-05 |
| 8 | sdcC02 | 123 | 24722 | 6.79E-23 |  | 42 | I95 | 18 | 1597 | 1.45E-02 |
| 8 | sdcC03 | 166 | 18771 | 2.90E-86 |  | 42 | L89 | 9 | 322 | 1.04E-02 |
| 8 | sdcC07 | 15 | 199 | 3.31E-04 |  | 42 | L97 | 24 | 1718 | 1.47E-07 |
| 8 | sdcC17 | 37 | 2472 | 9.91E-03 |  | 42 | M10 | 7 | 328 | 4.89E-02 |
| 8 | sdcC18 | 97 | 9919 | 2.08E-32 |  | 42 | M14 | 6 | 87 | 3.66E-02 |
| 8 | sdcC19 | 46 | 3495 | 5.04E-04 |  | 42 | N05 | 2 | 1 | 1.15E-02 |
| 8 | sdcC20 | 28 | 560 | 9.73E-10 |  | 42 | N18 | 5 | 16 | 1.79E-04 |
| 8 | sdcC33 | 77 | 7180 | 7.19E-20 |  | 42 | R23 | 31 | 4082 | 2.64E-06 |
| 8 | sdcC36 | 53 | 3871 | 2.46E-11 |  | 42 | R42 | 34 | 7046 | 1.67E-02 |
| 8 | sdcC37 | 25 | 638 | 1.34E-08 |  | 42 | R80 | 30 | 4986 | 1.49E-02 |
| 8 | sdcN11 | 132 | 48889 | 3.51E-02 |  | 42 | S82 | 4 | 12 | 1.71E-02 |
| 9 | E10 | 101 | 31168 | 1.24E-10 |  | 42 | Z89 | 18 | 417 | 1.87E-11 |
| 9 | I10 | 106 | 54934 | 1.96E-02 |  | 42 | Z94 | 84 | 950 | 3.95E-154 |
| 9 | R03 | 190 | 13194 | 2.89E-222 |  | 42 | sdcC17 | 15 | 693 | 9.63E-04 |
| 9 | sdcC12 | 149 | 75851 | 4.34E-18 |  | 42 | sdcC33 | 14 | 1122 | 2.24E-02 |
| 9 | sdcC14 | 132 | 78677 | 1.30E-04 |  | 42 | sdcE02 | 11 | 84 | 5.59E-07 |
| 9 | sdcE01 | 106 | 47332 | 5.64E-03 |  | 42 | sdcE04 | 32 | 5649 | 1.48E-04 |
| 9 | sdcE09 | 101 | 45011 | 3.51E-02 |  | 42 | sdcE05 | 17 | 806 | 1.78E-03 |
| 9 | sdcF10 | 30 | 2406 | 3.13E-03 |  | 42 | sdcE07 | 32 | 4505 | 1.11E-04 |
| 9 | sdcF15 | 100 | 42932 | 1.44E-02 |  | 42 | sdcE08 | 48 | 5233 | 1.04E-19 |
| 9 | sdcL05 | 153 | 92208 | 1.44E-09 |  | 42 | sdcE09 | 54 | 19808 | 3.31E-04 |
| 9 | sdcL09 | 112 | 45643 | 4.05E-06 |  | 42 | sdcE13 | 7 | 63 | 6.50E-04 |
| 9 | sdcM021 | 138 | 65281 | 5.39E-12 |  | 42 | sdcF02 | 25 | 2328 | 4.02E-06 |
| 9 | sdcM031 | 158 | 105661 | 9.09E-07 |  | 42 | sdcF04 | 33 | 3653 | 2.26E-09 |
| 9 | sdcM101 | 146 | 93678 | 2.80E-04 |  | 42 | sdcF05 | 51 | 10517 | 2.88E-11 |
| 9 | sdcM151 | 105 | 37986 | 1.46E-06 |  | 42 | sdcF18 | 27 | 2299 | 8.49E-09 |
| 9 | sdcM170 | 25 | 1376 | 6.48E-04 |  | 42 | sdcF19 | 21 | 2248 | 4.70E-03 |
| 9 | sdcO090 | 66 | 15775 | 3.04E-02 |  | 42 | sdcF21 | 12 | 657 | 1.37E-02 |
| 9 | sdcO17 | 190 | 13156 | 2.65E-222 |  | 42 | sdcK05 | 59 | 8410 | 4.23E-27 |
| 10 | E14 | 173 | 31084 | 5.10E-118 |  | 42 | sdcK06 | 31 | 1612 | 4.32E-14 |
| 10 | sdcO15 | 22 | 1307 | 2.07E-02 |  | 42 | sdcK07 | 28 | 1233 | 6.66E-14 |
| 11 | A02 | 2 | 1 | 2.85E-02 |  | 42 | sdcK08 | 61 | 15560 | 1.29E-14 |
| 11 | C85 | 3 | 3 | 1.42E-03 |  | 42 | sdcK12 | 36 | 956 | 7.02E-31 |
| 11 | E13 | 12 | 176 | 5.15E-04 |  | 42 | sdcM021 | 60 | 24371 | 3.37E-04 |
| 11 | E53 | 4 | 12 | 7.26E-03 |  | 42 | sdcN10 | 24 | 1916 | 5.08E-07 |
| 11 | E83 | 160 | 4879 | 2.24E-214 |  | 42 | sdcN11 | 49 | 18908 | 4.21E-03 |
| 11 | L73 | 3 | 3 | 5.98E-03 |  | 42 | sdcN12 | 39 | 11572 | 6.93E-03 |
| 12 | J44 | 8 | 72 | 2.22E-03 |  | 42 | sdcN13 | 25 | 2747 | 1.56E-05 |
| 12 | J45 | 156 | 6226 | 3.06E-199 |  | 42 | sdcO04 | 20 | 2075 | 7.11E-03 |
| 12 | R05 | 49 | 7953 | 4.37E-04 |  | 43 | L60 | 83 | 2244 | 3.96E-126 |
| 12 | sdcO02 | 24 | 167 | 9.57E-22 |  | 43 | R23 | 26 | 3750 | 5.36E-03 |
| 13 | R42 | 62 | 14739 | 7.11E-04 |  | 43 | sdcF04 | 23 | 3046 | 1.07E-02 |
| 13 | R51 | 147 | 24918 | 1.38E-92 |  | 43 | sdcF05 | 34 | 7903 | 1.16E-02 |
| 13 | VRA | 7 | 18 | 3.89E-05 |  | 44 | R45 | 81 | 18438 | 1.73E-40 |
| 14 | E10 | 72 | 22149 | 4.79E-05 |  | 45 | E11 | 62 | 23463 | 7.14E-05 |
| 14 | L84 | 144 | 14210 | 1.02E-136 |  | 45 | E66 | 19 | 1897 | 2.62E-02 |
| 14 | R73 | 110 | 64432 | 1.52E-02 |  | 45 | E87 | 27 | 2301 | 1.48E-05 |
| 14 | Z72 | 125 | 88377 | 1.54E-02 |  | 45 | I20 | 32 | 1605 | 5.32E-11 |
| 14 | sdcE01 | 75 | 30879 | 1.90E-02 |  | 45 | I21 | 9 | 116 | 3.65E-03 |
| 14 | sdcM021 | 97 | 42271 | 1.54E-05 |  | 45 | I25 | 32 | 2469 | 2.51E-10 |
| 14 | sdcM031 | 120 | 71425 | 3.06E-04 |  | 45 | I35 | 8 | 132 | 7.33E-03 |
| 14 | sdcM151 | 79 | 27423 | 8.68E-04 |  | 45 | I50 | 80 | 2957 | 1.28E-100 |
| 14 | sdcO090 | 59 | 14263 | 3.39E-02 |  | 45 | I95 | 27 | 2286 | 1.29E-04 |
| 15 | E10 | 64 | 19138 | 3.32E-04 |  | 45 | L89 | 11 | 338 | 4.42E-02 |
| 15 | L29 | 75 | 4344 | 4.03E-51 |  | 45 | R06 | 35 | 2692 | 2.75E-11 |
| 15 | M65 | 69 | 2357 | 4.38E-65 |  | 45 | R18 | 5 | 27 | 1.36E-02 |
| 15 | M72 | 8 | 122 | 7.98E-03 |  | 45 | Z95 | 6 | 42 | 2.43E-03 |
| 15 | R23 | 41 | 6481 | 6.12E-05 |  | 45 | sdcC02 | 44 | 7139 | 1.95E-07 |
| 15 | R73 | 103 | 63834 | 1.04E-02 |  | 45 | sdcC03 | 32 | 2802 | 4.05E-06 |
| 15 | sdcN11 | 72 | 32495 | 3.40E-02 |  | 45 | sdcC07 | 7 | 83 | 2.25E-02 |
| 16 | E11 | 68 | 27086 | 7.49E-03 |  | 45 | sdcC18 | 36 | 2951 | 1.81E-11 |
| 16 | L30 | 17 | 569 | 4.56E-04 |  | 45 | sdcC20 | 12 | 161 | 1.04E-04 |
| 16 | L40 | 128 | 3235 | 2.11E-199 |  | 45 | sdcC33 | 29 | 2785 | 7.96E-07 |
| 16 | M07 | 10 | 27 | 1.33E-10 |  | 45 | sdcC35 | 18 | 779 | 2.78E-03 |
| 17 | E06 | 6 | 17 | 4.14E-06 |  | 45 | sdcC36 | 16 | 1142 | 4.23E-02 |
| 17 | K59 | 106 | 10412 | 1.22E-76 |  | 45 | sdcK08 | 48 | 13202 | 1.34E-03 |
| 17 | R42 | 52 | 14585 | 3.82E-02 |  | 45 | sdcK10 | 7 | 133 | 1.86E-02 |
| 17 | T78 | 18 | 57 | 8.64E-23 |  | 45 | sdcN13 | 33 | 5438 | 4.87E-02 |
| 17 | sdcC22 | 9 | 166 | 3.28E-02 |  | 46 | I95 | 26 | 2432 | 2.42E-05 |
| 17 | sdcN07 | 21 | 1643 | 2.59E-02 |  | 46 | R26 | 3 | 7 | 1.11E-03 |
| 18 | E86 | 13 | 280 | 1.71E-02 |  | 46 | R33 | 5 | 94 | 2.71E-02 |
| 18 | E87 | 124 | 10878 | 5.00E-116 |  | 46 | R42 | 79 | 17860 | 5.79E-41 |
| 18 | M81 | 5 | 48 | 1.70E-02 |  | 47 | B18 | 2 | 1 | 4.07E-02 |
| 18 | R00 | 25 | 2302 | 1.30E-02 |  | 47 | F20 | 76 | 652 | 8.01E-141 |
| 18 | R80 | 53 | 10118 | 4.16E-06 |  | 47 | L84 | 19 | 1957 | 8.83E-03 |
| 18 | Z60 | 12 | 222 | 3.30E-04 |  | 47 | R44 | 5 | 8 | 5.19E-06 |
| 18 | sdcC06 | 49 | 9031 | 7.15E-04 |  | 47 | T73 | 40 | 9038 | 1.99E-04 |
| 18 | sdcC28 | 40 | 6568 | 4.13E-03 |  | 47 | Z72 | 68 | 40381 | 3.83E-02 |
| 18 | sdcC31 | 19 | 1196 | 8.59E-03 |  | 47 | sdcL01 | 30 | 5615 | 8.24E-03 |
| 18 | sdcC35 | 23 | 1683 | 6.58E-04 |  | 47 | sdcL02 | 14 | 816 | 2.62E-02 |
| 18 | sdcE04 | 37 | 6256 | 3.28E-02 |  | 48 | R06 | 77 | 7080 | 8.64E-76 |
| 18 | sdcE07 | 37 | 5649 | 9.65E-03 |  | 48 | sdcC02 | 29 | 6466 | 1.48E-02 |
| 18 | sdcE09 | 75 | 28494 | 1.14E-03 |  | 48 | sdcC36 | 16 | 1361 | 5.23E-03 |
| 18 | sdcF15 | 68 | 23034 | 3.35E-02 |  | 49 | E10 | 33 | 8413 | 3.50E-02 |
| 18 | sdcK02 | 49 | 6106 | 1.14E-09 |  | 49 | E15 | 74 | 1718 | 9.98E-98 |
| 18 | sdcK03 | 65 | 21500 | 1.81E-02 |  | 49 | T38 | 9 | 232 | 1.13E-02 |
| 18 | sdcK05 | 58 | 9319 | 4.43E-12 |  | 49 | sdcM021 | 49 | 16810 | 5.25E-03 |
| 18 | sdcK06 | 22 | 1431 | 9.65E-03 |  | 49 | sdcM031 | 67 | 30061 | 7.28E-04 |
| 18 | sdcK08 | 74 | 20932 | 6.66E-07 |  | 49 | sdcM101 | 59 | 25796 | 3.08E-02 |
| 18 | sdcK11 | 14 | 526 | 7.93E-03 |  | 49 | sdcM132 | 32 | 967 | 6.02E-22 |
| 18 | sdcK12 | 10 | 157 | 3.14E-02 |  | 49 | sdcM151 | 59 | 15195 | 1.81E-11 |
| 18 | sdcM042 | 21 | 1195 | 7.19E-03 |  | 49 | sdcO03 | 5 | 38 | 4.61E-02 |
| 18 | sdcN11 | 74 | 28644 | 3.26E-02 |  | 50 | E66 | 16 | 2123 | 3.72E-02 |
| 18 | sdcN12 | 61 | 19264 | 3.61E-02 |  | 50 | R35 | 18 | 1938 | 2.73E-03 |
| 18 | sdcO11 | 9 | 34 | 1.71E-06 |  | 50 | R39 | 69 | 7221 | 4.76E-73 |
| 19 | H40 | 94 | 2057 | 6.77E-114 |  | 51 | M06 | 6 | 61 | 2.26E-04 |
| 19 | H42 | 5 | 9 | 1.14E-04 |  | 51 | M25 | 67 | 2119 | 7.53E-97 |
| 19 | I64 | 28 | 1056 | 2.71E-13 |  | 51 | R52 | 49 | 24104 | 3.87E-03 |
| 19 | sdcE05 | 27 | 2152 | 1.55E-02 |  | 52 | C20 | 5 | 9 | 2.20E-07 |
| 20 | I10 | 67 | 30090 | 4.00E-02 |  | 52 | I45 | 60 | 1541 | 4.18E-85 |
| 20 | R05 | 118 | 20479 | 2.12E-85 |  | 53 | E10 | 45 | 14778 | 2.41E-09 |
| 20 | sdcC12 | 70 | 31958 | 2.48E-02 |  | 53 | M85 | 63 | 2080 | 1.93E-94 |
| 20 | sdcC14 | 82 | 43173 | 2.22E-03 |  | 53 | Z00 | 10 | 398 | 1.31E-03 |
| 21 | A46 | 105 | 3032 | 1.78E-134 |  | 53 | sdcM021 | 42 | 20617 | 2.48E-02 |
| 21 | E11 | 73 | 30193 | 2.06E-02 |  | 53 | sdcN11 | 55 | 26287 | 8.91E-12 |
| 21 | E66 | 29 | 2975 | 5.29E-04 |  | 53 | sdcN12 | 53 | 19342 | 9.12E-16 |
| 21 | E87 | 28 | 2833 | 4.40E-03 |  | 54 | K30 | 8 | 424 | 4.19E-02 |
| 21 | E89 | 3 | 8 | 2.46E-02 |  | 54 | R12 | 61 | 2072 | 1.39E-87 |
| 21 | F10 | 5 | 53 | 4.50E-02 |  | 54 | R19 | 24 | 2189 | 2.41E-09 |
| 21 | H26 | 14 | 221 | 1.01E-05 |  | 55 | R52 | 60 | 26632 | 1.53E-19 |
| 21 | I73 | 20 | 1370 | 9.02E-03 |  | 55 | sdcF01 | 5 | 36 | 1.72E-03 |
| 21 | I83 | 22 | 610 | 1.08E-09 |  | 56 | N40 | 60 | 3952 | 1.29E-64 |
| 21 | L30 | 22 | 936 | 5.67E-07 |  | 57 | D69 | 4 | 2 | 9.38E-08 |
| 21 | L84 | 30 | 2493 | 2.36E-05 |  | 57 | J00 | 56 | 2011 | 1.35E-70 |
| 21 | L97 | 46 | 3659 | 3.69E-13 |  | 57 | sdcM11 | 6 | 63 | 4.41E-03 |
| 21 | M14 | 9 | 182 | 6.79E-03 |  | 58 | Z00 | 58 | 2045 | 2.06E-84 |
| 21 | O92 | 10 | 85 | 1.80E-05 |  | 59 | N30 | 55 | 1826 | 6.66E-67 |
| 21 | R06 | 30 | 3011 | 2.97E-04 |  | 60 | E10 | 31 | 6691 | 8.53E-04 |
| 21 | R23 | 80 | 12316 | 3.41E-31 |  | 60 | Z60 | 56 | 817 | 3.67E-92 |
| 21 | R52 | 89 | 39767 | 9.84E-06 |  | 61 | C61 | 53 | 800 | 2.01E-83 |
| 21 | R80 | 40 | 8633 | 4.35E-02 |  | 61 | C79 | 12 | 17 | 2.91E-17 |
| 21 | sdcE09 | 70 | 25882 | 9.52E-03 |  | 61 | T81 | 2 | 2 | 1.69E-02 |
| 21 | sdcF02 | 51 | 5353 | 5.52E-13 |  | 61 | VRA | 2 | 6 | 4.05E-02 |
| 21 | sdcF04 | 53 | 6407 | 2.72E-10 |  | 62 | K30 | 53 | 2150 | 3.08E-68 |
| 21 | sdcF05 | 71 | 15604 | 1.25E-13 |  | 62 | K42 | 3 | 13 | 1.06E-02 |
| 21 | sdcF19 | 56 | 6690 | 2.31E-17 |  | 63 | R31 | 53 | 766 | 1.05E-94 |
| 21 | sdcF21 | 31 | 1523 | 1.56E-09 |  | 63 | R32 | 2 | 1 | 5.92E-03 |
| 21 | sdcK02 | 35 | 4736 | 2.66E-03 |  | 63 | R80 | 29 | 6629 | 1.24E-05 |
| 21 | sdcK05 | 38 | 6164 | 7.69E-03 |  | 63 | sdcK02 | 17 | 2478 | 4.54E-02 |
| 21 | sdcK08 | 90 | 24965 | 8.52E-18 |  | 63 | sdcK05 | 20 | 3565 | 1.83E-02 |
| 21 | sdcL12 | 21 | 1836 | 1.12E-02 |  | 64 | G83 | 50 | 1304 | 8.90E-74 |
| 21 | sdcN12 | 67 | 23473 | 7.23E-04 |  | 64 | R00 | 13 | 1368 | 2.92E-02 |
| 21 | sdcN13 | 48 | 9242 | 7.70E-04 |  | 64 | R01 | 5 | 15 | 1.57E-04 |
| 22 | E11 | 81 | 32348 | 9.54E-05 |  | 64 | sdcF07 | 11 | 360 | 7.04E-05 |
| 22 | I73 | 110 | 6289 | 6.77E-114 |  | 65 | K80 | 54 | 890 | 3.99E-91 |
| 22 | L14 | 5 | 16 | 1.99E-04 |  | 66 | E10 | 25 | 5994 | 3.02E-02 |
| 22 | R52 | 82 | 33769 | 4.53E-03 |  | 66 | I25 | 12 | 1216 | 3.82E-02 |
| 22 | R80 | 43 | 8844 | 4.63E-03 |  | 66 | I46 | 44 | 385 | 1.30E-77 |
| 22 | sdcC17 | 22 | 1408 | 3.48E-02 |  | 66 | I48 | 10 | 836 | 4.24E-02 |
| 22 | sdcC19 | 24 | 2461 | 2.96E-02 |  | 66 | M02 | 10 | 30 | 2.61E-14 |
| 22 | sdcC33 | 35 | 2937 | 2.25E-07 |  | 66 | M14 | 6 | 98 | 6.91E-03 |
| 22 | sdcF09 | 9 | 190 | 8.59E-03 |  | 66 | sdcC02 | 22 | 4201 | 2.32E-02 |
| 22 | sdcF15 | 111 | 37239 | 1.68E-34 |  | 66 | sdcE08 | 15 | 1491 | 3.26E-02 |
| 22 | sdcF17 | 7 | 35 | 1.27E-03 |  | 66 | sdcF05 | 24 | 4564 | 3.81E-02 |
| 22 | sdcN12 | 67 | 21740 | 7.44E-04 |  | 66 | sdcK03 | 33 | 10277 | 1.09E-02 |
| 23 | A46 | 11 | 277 | 8.58E-03 |  | 67 | I70 | 5 | 107 | 4.54E-02 |
| 23 | I83 | 15 | 396 | 9.01E-06 |  | 67 | M60 | 2 | 2 | 2.07E-02 |
| 23 | L29 | 21 | 1055 | 4.29E-05 |  | 67 | M79 | 52 | 971 | 2.61E-84 |
| 23 | L30 | 108 | 3916 | 3.19E-143 |  | 67 | R52 | 42 | 20323 | 4.40E-03 |
| 23 | L97 | 28 | 2853 | 8.14E-05 |  | 68 | I95 | 52 | 5354 | 4.31E-47 |
| 23 | R23 | 43 | 6483 | 3.99E-07 |  | 68 | R42 | 30 | 7479 | 5.73E-04 |
| 23 | sdcF02 | 31 | 3752 | 2.81E-04 |  | 69 | B03 | 50 | 882 | 4.77E-83 |
| 23 | sdcF04 | 38 | 4275 | 6.85E-06 |  | 69 | I24 | 2 | 5 | 1.49E-02 |
| 23 | sdcF12 | 7 | 103 | 2.80E-02 |  | 70 | R73 | 51 | 19753 | 4.90E-09 |
| 23 | sdcF19 | 26 | 2997 | 1.78E-02 |  | 70 | Z72 | 51 | 23369 | 1.71E-06 |
| 23 | sdcF21 | 20 | 1099 | 2.96E-04 |  | 71 | C50 | 43 | 884 | 1.29E-61 |
| 23 | sdcK08 | 54 | 13886 | 4.22E-03 |  | 71 | C78 | 3 | 3 | 7.47E-04 |
| 24 | E87 | 20 | 1939 | 2.19E-02 |  | 71 | C79 | 5 | 8 | 2.12E-06 |
| 24 | G40 | 108 | 1323 | 7.63E-186 |  | 71 | K76 | 7 | 20 | 1.39E-09 |
