# Supplementary material for: Linking glycemic dysregulation in diabetes to symptoms, comorbidities, and genetics through EHR data mining
Source: eLife. 2019 Dec 10;8:e44941. doi: 10.7554/eLife.44941 (PMC6904221; doi:10.7554/eLife.44941)
Supplement: Supplementary file 5. [file elife-44941-supp5.docx]

**Supplementary Materials**

**Kirk and Simon et al.,**

**Linking glycemic dysregulation in diabetes to symptoms, comorbidities and genetics through EHR data mining.**

**Supplementary Table 5. The five top ranked independent genetic associations for individual clusters, symptom clusters, and dysregulation.** The five highest ranked (p-value) independent signals for each cluster/grouping. Positions are GRCh37. Effect is odds ratio for all analysis apart from “Dysregulation” which is treated as a quantitative trait (a score ranking from 0 (low) to 5 (high) based on number of dysregulation parameters). Here the effect is the beta estimate. All analyses are adjusted for age, sex and principal components. Nearest gene is assigned if a gene is present within +/- 25.000 base pairs from the SNP. A GWAS trait is mapped based on lookup using the nearest gene. If no genes can be mapped any GWAS signal within the +/- 25.000 base pair boundary is reported. For some of the genes only a selection of GWAS traits are included.

| **Grouping** | **rs ID** | **Chr** | **Position** | **Effect** | **P** | **MAF** | **Nearest gene** | **Mapped GWAS trait** |
| --- | --- | --- | --- | --- | --- | --- | --- | --- |
| Cluster 1 | rs74942512 | 4 | 84897265 | 5.4 | 4.14E-07 | 0.01 | *LOC101928978* |  |
| Cluster 1 | rs4323359 | 6 | 8913079 | 1.6 | 5.96E-07 | 0.34 |  |  |
| Cluster 1 | rs6603786 | 1 | 1187993 | 2.2 | 7.53E-07 | 0.07 | *UBE2J2* | Inflammatory bowel disease, Ulcerative colitis |
| Cluster 1 | rs74562926 | 1 | 37009206 | 2.1 | 9.84E-07 | 0.09 |  |  |
| Cluster 1 | rs17074389 | 8 | 5506702 | 1.6 | 1.18E-06 | 0.34 |  |  |
| Cluster 2 | rs117805124 | 6 | 130998382 | 19.9 | 5.01E-09 | 0.01 |  |  |
| Cluster 2 | rs61743921 | 1 | 201184878 | 7.0 | 4.02E-08 | 0.06 | *IGFN1* |  |
| Cluster 2 | rs78062255 | 2 | 66018309 | 11.8 | 3.37E-07 | 0.01 | *FLJ16124* | Pulse pressure in young-onset hypertension, Post bronchodilator FEV1 |
| Cluster 2 | rs17130038 | 1 | 83964417 | 10.3 | 4.52E-07 | 0.02 |  |  |
| Cluster 2 | rs115012219 | 3 | 5820451 | 7.2 | 4.57E-07 | 0.03 |  |  |
| Cluster 3 | rs138250961 | 10 | 103318560 | 5.7 | 7.89E-08 | 0.02 | *BTRC* |  |
| Cluster 3 | rs117046925 | 4 | 101228459 | 6.8 | 2.57E-07 | 0.02 |  |  |
| Cluster 3 | rs113321747 | 13 | 104838591 | 6.1 | 3.51E-07 | 0.02 |  |  |
| Cluster 3 | rs2830783 | 21 | 28584349 | 2.3 | 5.79E-07 | 0.18 |  |  |
| Cluster 3 | rs74634742 | 2 | 171386194 | 3.3 | 7.94E-07 | 0.05 | *MYO3B* | Obesity related traits, Visceral fat |
| Cluster 4 | rs62387060 | 5 | 99491831 | 10.4 | 9.16E-10 | 0.02 |  |  |
| Cluster 4 | rs116598593 | 2 | 224896162 | 10.9 | 7.05E-08 | 0.01 | *SERPINE2* | Interleukin 2, 6, 10 levels, Platelet-derived growth factor BB levels |
| Cluster 4 | rs144128923 | 9 | 90280614 | 10.8 | 2.38E-07 | 0.01 | *DAPK1* | Blood pressure, Bipolar disorder and schizophrenia |
| Cluster 4 | rs71452661 | 12 | 119080430 | 10.4 | 3.67E-07 | 0.01 | *LINC02460* |  |
| Cluster 4 | rs6587312 | 22 | 49137795 | 3.5 | 4.51E-07 | 0.18 | *FAM19A5* | Pancreatic cancer, Menarche (age at onset) |
| Cluster 5 | rs117653734 | 14 | 97804501 | 5.5 | 3.08E-08 | 0.02 |  |  |
| Cluster 5 | rs282053 | 1 | 91824625 | 2.7 | 6.15E-08 | 0.08 | *HFM1* | C-reactive protein levels or total cholesterol levels, Metabolic syndrome |
| Cluster 5 | rs2298541 | 18 | 3010450 | 3.8 | 5.22E-07 | 0.03 | *LPIN2* | Type 2 diabetes, Body mass index in asthmatics, Urinary albumin-to-creatinine ratio in non-diabetics |
| Cluster 5 | rs76714803 | 6 | 73250607 | 3.3 | 5.59E-07 | 0.03 |  |  |
| Cluster 5 | rs142854195 | 8 | 28257804 | 4.2 | 5.71E-07 | 0.02 | *ZNF395* |  |
| Cluster 7 | rs188511762 | 1 | 83102286 | 10.3 | 1.64E-08 | 0.02 |  |  |
| Cluster 7 | rs147915743 | 4 | 188479676 | 12.7 | 1.67E-07 | 0.01 | *LINC02492* |  |
| Cluster 7 | rs117608019 | 9 | 79061435 | 8.3 | 1.85E-07 | 0.04 | *GCNT1* | Post bronchodilator FEV1/FVC ratio in COPD, Dialysis-related mortality |
| Cluster 7 | rs10483637 | 14 | 55157430 | 7.8 | 2.69E-07 | 0.06 | *SAMD4A* | Obesity related traits, Lung function |
| Cluster 7 | rs116697536 | 3 | 60602727 | 8.7 | 2.78E-07 | 0.02 | *FHIT* | BMI, Photic sneeze reflex |
| Cluster 8 | rs73445432 | 12 | 130546874 | 6.7 | 1.88E-08 | 0.03 | *LINC02419* |  |
| Cluster 8 | rs56310054 | 5 | 79307250 | 8.4 | 4.88E-08 | 0.02 | *THBS4* | LDL peak particle diameter, Serum thyroid-stimulating hormone levels |
| Cluster 8 | rs151250741 | 17 | 50939500 | 6.0 | 9.10E-08 | 0.02 | *LINC02089* |  |
| Cluster 8 | rs62219570 | 21 | 39852079 | 5.3 | 9.55E-08 | 0.05 | *ERG* | White blood cell types, Abdominal aortic aneurysm, Pulse pressure |
| Cluster 8 | rs73084384 | 20 | 8262090 | 9.3 | 1.19E-07 | 0.01 | *PLCB1* | Diastolic blood pressure |
| Cluster 9 | rs114171886 | 3 | 77851724 | 12.2 | 3.05E-09 | 0.01 |  |  |
| Cluster 9 | rs73153368 | 20 | 54756509 | 8.4 | 9.16E-09 | 0.02 | *RNA5SP487* |  |
| Cluster 9 | rs7200108 | 16 | 13438884 | 4.1 | 2.48E-07 | 0.10 | *U91319.1* | Schizophrenia |
| Cluster 9 | rs74557062 | 9 | 78087123 | 4.3 | 2.76E-07 | 0.08 |  |  |
| Cluster 9 | rs76021855 | 14 | 98856704 | 7.0 | 4.81E-07 | 0.02 |  |  |
| Cluster 12 | rs188798945 | 5 | 102092967 | 18.8 | 1.54E-09 | 0.01 | *PAM* | Primary biliary cholangitis |
| Cluster 12 | rs10209611 | 2 | 1995286 | 14.6 | 1.67E-08 | 0.02 | *MYT1L* | Cognitive decline rate in late mild cognitive impairment, Glucose Homeostasis Traits |
| Cluster 12 | rs116007420 | 3 | 31919004 | 11.4 | 4.63E-08 | 0.02 | *OSBPL10* | Peripheral artery disease, Periodontal disease-related phenotypes |
| Cluster 12 | rs2283324 | 12 | 2646485 | 14.8 | 6.65E-08 | 0.01 | *CACNA1C* | Post bronchodilator FEV1/FVC ratio, Schizophrenia |
| Cluster 12 | rs11841622 | 13 | 22769891 | 5.1 | 1.64E-07 | 0.11 | *LINC00540* | Abdominal aortic aneurysm, Adverse response to chemotherapy |
| Cluster 15 | rs148761875 | 17 | 55840408 | 13.0 | 2.01E-08 | 0.01 | *CCDC182* | Schizophrenia, Sex hormone-binding globulin levels |
| Cluster 15 | rs35156277 | 14 | 105510475 | 5.2 | 2.61E-08 | 0.07 | *GPR132* |  |
| Cluster 15 | rs117235948 | 8 | 77385117 | 5.7 | 1.40E-07 | 0.05 | *LINC01111* | Ovarian cancer |
| Cluster 15 | rs199528724 | 16 | 88504203 | 9.1 | 1.44E-07 | 0.01 | *ZNF469* | Corneal structure, Central corneal thickness |
| Cluster 15 | rs116806515 | 1 | 96887339 | 9.9 | 5.01E-07 | 0.01 | *UBE2WP1* | BMI, Hip circumference, Waist circumference |
| Cluster 16 | rs79933215 | 10 | 1498982 | 9.9 | 1.95E-08 | 0.04 | *ADARB2* | Blood pressure, Migraine, Menarche |
| Cluster 16 | rs74802033 | 16 | 11334171 | 7.2 | 1.29E-07 | 0.06 | *HNRNPCP4* |  |
| Cluster 16 | rs62397613 | 6 | 20645736 | 10.1 | 5.88E-07 | 0.02 | *CDKAL1* | Type 2 diabetes, BMI |
| Cluster 16 | rs139445702 | 12 | 122862233 | 15.2 | 8.04E-07 | 0.01 | *CLIP1* | BMI, Hip circumference, Schizophrenia |
| Cluster 16 | rs113131193 | 2 | 23490018 | 6.5 | 1.07E-06 | 0.07 |  |  |
| Cluster 17 | rs75190244 | 8 | 96287633 | 10.3 | 2.56E-08 | 0.03 | *C8orf37-AS1* | Obesity-related traits |
| Cluster 17 | rs113190554 | 6 | 97253024 | 12.0 | 1.74E-07 | 0.02 | *GPR63* |  |
| Cluster 17 | rs73185345 | 3 | 178633612 | 13.5 | 2.13E-07 | 0.01 |  |  |
| Cluster 17 | rs145554678 | 8 | 27118107 | 11.5 | 2.25E-07 | 0.01 | *STMN4* | Bipolar disorder and schizophrenia |
| Cluster 17 | rs112136315 | 8 | 94722725 | 9.5 | 2.64E-07 | 0.03 | *FAM92A* |  |
| Cluster 18 | rs71429242 | 2 | 220759984 | 9.0 | 4.12E-08 | 0.03 | *LINC01803* |  |
| Cluster 18 | rs115036356 | 3 | 13191877 | 11.8 | 6.41E-08 | 0.01 |  |  |
| Cluster 18 | rs138967674 | 6 | 56434712 | 12.5 | 2.02E-07 | 0.01 | *DST* | Menarche, ADHD, Schizophrenia |
| Cluster 18 | rs75158140 | 17 | 13516302 | 7.2 | 2.11E-07 | 0.04 | *HS3ST3A1* | Cognitive decline rate in late mild cognitive impairment |
| Cluster 18 | rs141703713 | 16 | 51842233 | 7.1 | 2.19E-07 | 0.03 |  |  |
| Cluster 21 | rs17051344 | 4 | 122285263 | 4.3 | 2.08E-08 | 0.09 | *QRFPR* |  |
| Cluster 21 | rs75344826 | 18 | 33896157 | 9.0 | 3.58E-08 | 0.02 | *FHOD3* | Resting heart rate, QRS duration, Obesity-related traits |
| Cluster 21 | rs143872989 | 1 | 179411175 | 8.8 | 3.13E-07 | 0.02 | *AXDND1* | Multiple sclerosis (age of onset) |
| Cluster 21 | rs12416742 | 11 | 18459126 | 5.0 | 4.16E-07 | 0.04 | *LDHC* | Amyloid A serum levels, Blood protein levels |
| Cluster 21 | rs75367635 | 13 | 113748555 | 7.2 | 5.06E-07 | 0.02 | *MCF2L* | Systolic blood pressure, End-stage coagulation, Prothrombin time |
| Cluster 22 | rs146003545 | 9 | 110942792 | 12.8 | 1.14E-08 | 0.01 |  |  |
| Cluster 22 | rs35322693 | 17 | 5766059 | 7.5 | 3.31E-08 | 0.03 | *WSCD1* | Migraine, Menarche, Cognitive decline rate |
| Cluster 22 | rs1928333 | 1 | 104732432 | 6.0 | 2.12E-07 | 0.03 | *AC092506.1* |  |
| Cluster 22 | rs113187799 | 7 | 113008872 | 6.7 | 5.54E-07 | 0.02 |  |  |
| Cluster 22 | rs62414179 | 6 | 93901681 | 6.9 | 8.15E-07 | 0.02 |  |  |
| Cluster 23 | rs114781132 | 1 | 12612552 | 10.7 | 3.05E-08 | 0.02 | *DHRS3* | Optic cup area |
| Cluster 23 | rs114712997 | 14 | 21342523 | 12.5 | 6.01E-08 | 0.02 |  |  |
| Cluster 23 | rs71631858 | 1 | 196608942 | 16.3 | 6.30E-08 | 0.01 | *CFH* | Age-related macular degeneration, Post bronchodilator FEV1/FVC ratio in COPD |
| Cluster 23 | rs149235083 | 11 | 57727600 | 11.0 | 1.28E-07 | 0.02 | *OR5BD1P* | Parkinson's disease |
| Cluster 23 | rs117618853 | 12 | 81768580 | 13.2 | 1.31E-07 | 0.01 | *PPFIA2* | Resting heart rate, Age-related cataracts, Schizophrenia, Post bronchodilator FEV1 |
| Cluster 24 | rs12797453 | 11 | 27038907 | 20.3 | 7.07E-09 | 0.01 | *BBOX1* |  |
| Cluster 24 | rs75067583 | 13 | 112094648 | 13.7 | 1.45E-08 | 0.02 |  |  |
| Cluster 24 | rs151125779 | 18 | 8838774 | 14.1 | 3.16E-08 | 0.02 | *MTCL1* | COPD, Obesity related traits, Peripheral arterial disease |
| Cluster 24 | rs146152902 | 1 | 239244933 | 9.7 | 1.63E-07 | 0.03 |  |  |
| Cluster 24 | rs1497850 | 1 | 74256107 | 12.5 | 2.16E-07 | 0.01 |  |  |
| Cluster 26 | rs146300760 | 19 | 35012737 | 6.0 | 1.01E-08 | 0.05 | *WTIP* |  |
| Cluster 26 | rs149240739 | 15 | 63255898 | 7.6 | 1.55E-07 | 0.03 |  |  |
| Cluster 26 | rs74687760 | 5 | 110085877 | 13.3 | 2.37E-07 | 0.01 | *SLC25A46* | Asthma, Allergy, Mortality in heart failure |
| Cluster 26 | rs77929904 | 11 | 131751064 | 9.0 | 2.70E-07 | 0.02 | *NTM* | Food addiction, Educational attainment, Myopia, Obesity related traits |
| Cluster 26 | rs62409210 | 4 | 25412773 | 7.8 | 4.03E-07 | 0.02 | *ANAPC4* | Weight, Rheumatoid arthritis |
| Cluster 31 | rs4407469 | 4 | 94812586 | 7.1 | 8.29E-09 | 0.03 |  |  |
| Cluster 31 | rs61851672 | 10 | 57314616 | 5.6 | 7.40E-08 | 0.04 | *PCDH15* | Carotid artery intima media thickness, Cardiac hypertrophy |
| Cluster 31 | rs190029411 | 20 | 59904877 | 13.3 | 1.65E-07 | 0.01 | *CDH4* | Educational attainment, Obesity-related traits, Cognitive decline rate in late mild cognitive impairment |
| Cluster 31 | rs72884620 | 2 | 167852274 | 4.8 | 2.40E-07 | 0.06 | *XIRP2* | Atopic dermatitis, Interleukin 6 levels |
| Cluster 31 | rs72705934 | 4 | 183811385 | 7.7 | 3.45E-07 | 0.02 | *DCTD* | Obesity-related traits, Diisocyanate-induced asthma, Wegener's granulomatosis |
| Cluster 35 | rs78656858 | 13 | 74618033 | 8.0 | 8.30E-08 | 0.03 | *KLF12* | Type 2 diabetes, Glucose homeostasis traits, Sudden cardiac arrest, Schizophrenia |
| Cluster 35 | rs77369782 | 20 | 49039134 | 9.3 | 1.05E-07 | 0.03 |  |  |
| Cluster 35 | rs75345663 | 16 | 21338483 | 5.5 | 1.41E-07 | 0.06 | *CRYM-AS1* |  |
| Cluster 35 | rs117408261 | 17 | 80332067 | 9.7 | 1.80E-07 | 0.02 | *UTS2R* |  |
| Cluster 35 | rs117058170 | 16 | 28589373 | 12.6 | 2.20E-07 | 0.01 | *SGF29* |  |
| Cluster 39 | rs112851872 | 18 | 33868418 | 8.5 | 3.96E-08 | 0.07 | *FHOD3* | Resting heart rate, QRS duration, Obesity-related traits |
| Cluster 39 | rs9809270 | 3 | 142162680 | 14.8 | 7.73E-08 | 0.02 | *XRN1* |  |
| Cluster 39 | rs187557324 | 14 | 54580801 | 13.1 | 8.99E-08 | 0.02 |  |  |
| Cluster 39 | rs544250203 | 15 | 49838932 | 18.3 | 1.36E-07 | 0.01 | *FAM227B* | Thyroid volume, Thyroid hormone levels |
| Cluster 39 | rs145251891 | 19 | 58455076 | 14.2 | 2.69E-07 | 0.02 | *ZNF256* |  |
| Cluster 45 | rs7181631 | 15 | 99177802 | 7.4 | 1.68E-07 | 0.05 | *IGF1R* | Fasting plasma glucose, Height, Endometriosis |
| Cluster 45 | rs35258344 | 17 | 59360358 | 8.3 | 2.77E-07 | 0.04 | *BCAS3* | Glomerular filtration rate, Chronic kidney disease, Gout, Renal function, Coronary artery disease |
| Cluster 45 | rs7130813 | 11 | 78688826 | 7.6 | 3.64E-07 | 0.03 | *TENM4* | Bipolar disorder, Autism spectrum disorder, ADHD, Obesity related traits |
| Cluster 45 | rs192601723 | 22 | 32707386 | 17.7 | 3.90E-07 | 0.01 |  |  |
| Cluster 45 | rs1218423 | 10 | 14359316 | 17.3 | 6.35E-07 | 0.01 | *FRMD4A* | Alzheimer’s, Craniofacial microsomia, Obstetric antiphospholipid syndrome, Smoking behaviour, Peripheral arterial disease |
| Cluster 46 | rs78019176 | 4 | 110406970 | 25.6 | 3.03E-08 | 0.01 | *SEC24B* | Late-onset Alzheimer's disease |
| Cluster 46 | rs76086776 | 3 | 104722049 | 13.7 | 1.78E-07 | 0.02 |  |  |
| Cluster 46 | rs71413498 | 14 | 78840294 | 12.8 | 1.83E-07 | 0.04 | *NRXN3* | BMI, Hip circumference, Waist circumference, Cognitive performance |
| Cluster 46 | rs117739592 | 11 | 110145457 | 20.2 | 2.00E-07 | 0.01 | *RDX* | Cognitive function |
| Cluster 46 | rs111569944 | 9 | 106569243 | 16.9 | 2.08E-07 | 0.01 |  |  |
| Cluster 66 | rs9383810 | 6 | 156853634 | 21.2 | 5.57E-09 | 0.02 |  |  |
| Cluster 66 | rs62483864 | 8 | 510212 | 11.0 | 2.11E-07 | 0.02 | *C8orf42* | Bilirubin levels |
| Cluster 66 | rs141012001 | 16 | 26672005 | 17.2 | 2.26E-07 | 0.01 |  |  |
| Cluster 66 | rs113211483 | 5 | 123915576 | 11.9 | 2.30E-07 | 0.03 |  |  |
| Cluster 66 | rs1423276 | 5 | 91257092 | 8.4 | 2.56E-07 | 0.04 |  |  |
| Symptom group 2 | rs150406145 | 2 | 6371190 | 7.9 | 5.93E-10 | 0.01 |  |  |
| Symptom group 2 | rs138392129 | 4 | 15138084 | 8.0 | 1.36E-08 | 0.01 | *AC098829.1* |  |
| Symptom group 2 | rs147890857 | 2 | 3757511 | 5.9 | 5.54E-08 | 0.02 | *DCDC2C* | Obesity-related traits, Type 2 diabetes |
| Symptom group 2 | rs17270020 | 15 | 60440357 | 2.8 | 1.24E-07 | 0.19 |  |  |
| Symptom group 2 | rs115850844 | 2 | 209779214 | 4.1 | 3.07E-07 | 0.04 |  |  |
| Symptom group 3 | rs76790102 | 14 | 55569578 | 2.8 | 2.97E-08 | 0.08 |  |  |
| Symptom group 3 | rs12579436 | 12 | 83719018 | 3.4 | 3.66E-07 | 0.03 |  |  |
| Symptom group 3 | rs56044713 | 13 | 93285050 | 5.0 | 3.99E-07 | 0.01 | *GPC5* | Blood protein levels, Height, Metabolite levels, Nephrotic syndrome, Obesity related traits |
| Symptom group 3 | rs2881264 | 15 | 86771961 | 2.0 | 4.49E-07 | 0.22 | *AGBL1* | Activated partial thromboplastin time, Modified Stumvoll Insulin Sensitivity Index (BMI interaction), Ischemic stroke |
| Symptom group 3 | rs116169323 | 1 | 164902879 | 4.0 | 7.74E-07 | 0.02 |  |  |
| Symptom group 4 | rs58585758 | 7 | 36474413 | 4.9 | 3.65E-07 | 0.01 | *ANLN* | Systolic blood pressure, Obesity related traits |
| Symptom group 4 | rs2241585 | 5 | 175956516 | 1.4 | 1.37E-06 | 0.49 | *RNF44* | Menopause, Venous thromboembolism |
| Symptom group 4 | rs148724814 | 7 | 36390332 | 4.5 | 2.26E-06 | 0.01 | *KIAA0895* |  |
| Symptom group 4 | rs7678212 | 4 | 6592061 | 1.8 | 2.81E-06 | 0.07 | *MAN2B2* |  |
| Symptom group 4 | rs2116019 | 7 | 49346816 | 1.4 | 3.33E-06 | 0.37 |  |  |
| Symptom group 5 | rs137855175 | 2 | 199458046 | 3.9 | 1.76E-07 | 0.02 |  | Cognitive performance |
| Symptom group 5 | rs117691400 | 12 | 87329652 | 3.6 | 9.06E-07 | 0.02 |  | Post bronchodilator FEV1/FVC ratio |
| Symptom group 5 | rs67215596 | 12 | 32879543 | 1.9 | 9.92E-07 | 0.13 | *DNM1L* | Post bronchodilator FEV1 |
| Symptom group 5 | rs138931689 | 1 | 87879265 | 2.3 | 1.34E-06 | 0.05 |  | Resting heart rate |
| Symptom group 5 | rs76548985 | 13 | 85947423 | 2.7 | 1.43E-06 | 0.03 | *LINC00351* | Amyotrophic lateral sclerosis (sporadic), Aluminium levels |
| Symptom group 6 | rs28735051 | 15 | 92296505 | 0.6 | 5.01E-08 | 0.39 |  |  |
| Symptom group 6 | rs6823240 | 4 | 185809166 | 2.5 | 2.68E-06 | 0.03 | *LINC01093* |  |
| Symptom group 6 | rs73179511 | 22 | 49207926 | 1.6 | 3.05E-06 | 0.16 | *FAM19A5* | Pancreatic cancer, Menarche (age at onset) |
| Symptom group 6 | rs2500209 | 6 | 77357617 | 0.4 | 3.32E-06 | 0.09 |  |  |
| Symptom group 6 | rs6084855 | 20 | 4728248 | 0.6 | 3.60E-06 | 0.23 | *PRNT* | Systemic lupus erythematosus |
| Symptom group 7 | rs7638118 | 3 | 186974026 | 2.1 | 2.69E-07 | 0.12 | *MASP1* | Amyotrophic lateral sclerosis (sporadic) |
| Symptom group 7 | rs77601943 | 3 | 32889098 | 4.6 | 3.03E-07 | 0.02 | *TRIM71* | Pulmonary function, Venous thromboembolism |
| Symptom group 7 | rs6590490 | 11 | 130533026 | 1.8 | 3.14E-07 | 0.29 | *MIR8052* | Pulse pressure, Urate levels in lean individuals, Periodontitis |
| Symptom group 7 | rs35307190 | 7 | 76984290 | 2.8 | 4.93E-07 | 0.04 | *GSAP* | Clozapine-induced agranulocytosis/granulocytopenia in treatment-resistant schizophrenia |
| Symptom group 7 | rs55818284 | 9 | 84188660 | 2.7 | 5.26E-07 | 0.04 | *TLE1* | Type 2 diabetes, Schizophrenia, Visceral adipos tissue adjusted for BMI |
| Dysregulation | rs1155820 | 2 | 133613920 | -0.2 | 1.46E-07 | 0.26 | *NCKAP5* | Glaucoma, Bipolar disorder and schizophrenia, ADHD |
| Dysregulation | rs2720371 | 4 | 10617751 | -0.2 | 4.31E-07 | 0.20 | *CLNK* | Myopia, Rheumatoid arthritis, Vitiligo |
| Dysregulation | rs17127410 | 8 | 18749120 | 0.2 | 5.38E-07 | 0.13 | *PSD3* | Metabolic traits, blood metabolite levels |
| Dysregulation | rs117165579 | 6 | 117012261 | -0.3 | 1.19E-06 | 0.06 | *KPNA5* | Telomere length |
| Dysregulation | rs140137433 | 13 | 84762770 | -0.4 | 1.93E-06 | 0.03 | *LINC00333* | Schizophrenia, Coronary heart disease, Obesity related traits, Temperament, Age-related hearing impairment |
