# Supplementary material for: Linking glycemic dysregulation in diabetes to symptoms, comorbidities, and genetics through EHR data mining
Source: eLife. 2019 Dec 10;8:e44941. doi: 10.7554/eLife.44941 (PMC6904221; doi:10.7554/eLife.44941)
Supplement: Supplementary file 6. [file elife-44941-supp6.docx]

**Supplementary Materials**

**Kirk and Simon et al.,**

**Linking glycemic dysregulation in diabetes to symptoms, comorbidities and genetics through EHR data mining.**

**Supplementary Table 6. The SDC-custom dictionary**. This vocabulary was created by the physicians at SDCC. Each clinical term is divided into the groups symptom, lifestyle (L), diabetes subtype (S), and treatment (T). The symptom group is further divided into complication areas: cardiovascular (C), eyes (E), feet related (F), kidneys (K), metabolic (M), neurological (N), and others (O). The table is color coded according to these groups.

| **Symptom** | **Name** | **Symptom** | **Name** | **Lifestyle** | **Name** |
| --- | --- | --- | --- | --- | --- |
| sdcM01.2 | diabetic coma | sdcE01 | retinal disorders | sdcL01 | Never alcohol |
| sdcM02.1 | hypoglycemia observation | sdcE02 | blindness | sdcL02 | Poor compliance |
| sdcM03.1 | hypoglycemia | sdcE03 | vitreous | sdcL03 | Dysregulated |
| sdcM04.2 | ketoacidose | sdcE04 | maculopathy | sdcL04 | Good compliance |
| sdcM06.0 | no hypoglycemia observation | sdcE05 | low visus | sdcL05 | Non-smoker |
| sdcM07 | elektrolytforstyrrelse | sdcE06 | retinal detachment | sdcL06 | Exercising, never |
| sdcM08 | honeymoon | sdcE07 | eye complications | sdcL07 | Aware of correct eating |
| sdcM09.1 | hypoglycemia medicamentalis | sdcE08 | proliferative retinopathy | sdcL08 | Communication problems |
| sdcM10.1 | hypoglycemia | sdcE09 | retinopathy | sdcL09 | Exercising, daily |
| sdcM11 | no congestion of adipose tissue | sdcE10 | retinopathy grade 1 | sdcL10 | Exercising, a lot |
| sdcM12 | injektionsinfiltrat | sdcE11 | retinopathy grade 2 | sdcL11 | Exercing, rarely |
| sdcM13.2 | insulin chock | sdcE12 | retinopathy grade 3 | sdcL12 | Obesity |
| sdcM14 | congestion of adipose tissue | sdcE13 | retinopathy grade 4 | **Diabetes subtype** | **Name** |
| sdcM15.1 | hypoglycemia observation | sdcF01 | varicose veins | sdcS1 | Maturity Onset Diabetes of the Young Type 1 |
| sdcM15.1 | it hypoglycemia observation | sdcF02 | foot ulcers | sdcS2 | Maturity Onset Diabetes of the Young Type 2 |
| sdcM17.0 | rare hypoglycemia observation | sdcF03 | gangrene | sdcS3 | Maturity Onset Diabetes of the Young Type 3 |
| sdcM18 | gastric bypass | sdcF04 | holstein | sdcS4 | Gestationel diabetes mellitus |
| sdcM19 | 4-metabolic treatment | sdcF05 | infection | sdcS5 | Latent Autoimmune Diabetes of Adults |
| sdcM20 | antidiabetics | sdcF06 | cold feet | **Treatment** | **Name** |
| sdcC01 | acute coronary syndrome | sdcF07 | mrsa | sdcT01 | 4-metabolic treatment |
| sdcC02 | AMI | sdcF08 | multi-resistant bacteria | sdcT02 | ACE antagonist |
| sdcC03 | angina | sdcF09 | loss of foot puls | sdcT03 | Amputation |
| sdcC04 | anterior infarction | sdcF10 | palpable foot puls | sdcT04 | Antibiotics |
| sdcC05 | Anteroseptalt infarction | sdcF11 | recurrent infekcion | sdcT05 | Antidiabetics |
| sdcC06 | apoplexy | sdcF12 | wound on shin | sdcT06 | arb_test |
| sdcC07 | arteriosclerotic heart disease | sdcF13 | ulcus pedis | sdcT07 | at2-antagonist |
| sdcC08 | stroke | sdcF14 | Resting until pain stops | sdcT07 | at2-blocker |
| sdcC09 | heart attack | sdcF14 | Resting until pain stops | sdcT08 | Betablocker |
| sdcC10 | cerebral infarction | sdcF15 | atherosclerosis | sdcT09 | Blood pressure medicin |
| sdcC11 | cordis arteioscleroticus | sdcF16 | with angiopatia diabetica extremitatum | sdcT10 | Prosthesis for pants |
| sdcC12 | hypertension | sdcF17 | decreased walking distance | sdcT11 | Bypass |
| sdcC13 | stroke | sdcF18 | amputation | sdcT12 | Calcium antagonist |
| sdcC14 | hypertensio arterialis | sdcF19 | antibiotics | sdcT13 | Dialysis |
| sdcC14 | hypertension | sdcF20 | prosthesis for pants | sdcT14 | Diuretica |
| sdcC16 | infarction | sdcF21 | grafting | sdcT15 | Gastric bypass |
| sdcC17 | ischaemia | sdcF22 | grafting of ulcers | sdcT16 | Coronary arteriography |
| sdcC18 | ischemic heart disease | sdcK01 | low diuresis | sdcT17 | Pci angioplasty |
| sdcC19 | macroangiopathy | sdcK02 | ma macro albuminuria | sdcT18 | Swab |
| sdcC20 | morbus cordis arterioscleroticus | sdcK02 | macro albuminuria | sdcT19 | Renography |
| sdcC22 | lowered beat to beat variation | sdcK03 | micro albuminuria | sdcT20 | Inoculation |
| sdcC23 | necrobiosis | sdcK05 | nephropathy |  |  |
| sdcC24 | Stenocardia | sdcK06 | kidney complications |  |  |
| sdcC06 | stroke | sdcK07 | kidney disease |  |  |
| sdcC26 | transient ischemic attack | sdcK08 | edema |  |  |
| sdcC27 | vascular insufficiency | sdcK09 | high diuresis |  |  |
| sdcC28 | ACE antagonist | sdcK10 | edema |  |  |
| sdcC29 | at2-antagonist | sdcK11 | renography |  |  |
| sdcC30 | at2-blocker | sdcK12 | dialysis |  |  |
| sdcC31 | beta-blocker | sdcO01 | amnesic symptoms |  |  |
| sdcC32 | blood pressure medications | sdcO02 | asthma bronchiale |  |  |
| sdcC33 | bypass | sdcO03 | unconsciousness |  |  |
| sdcC34 | calcium antagonist | sdcO04 | bleeding |  |  |
| sdcC35 | diuretics | sdcO05 | comorbidities |  |  |
| sdcC36 | coronary arteriography | sdcO06 | gastroenteritis |  |  |
| sdcC37 | angioplasty | sdcO07 | high alat |  |  |
| sdcN01 | autonomic dysfunction | sdcO08.0 | no comorbidities |  |  |
| sdcN02 | dificulty emptying the bladder | sdcO09.0 | no complications |  |  |
| sdcN03 | erection difficulties | sdcO09.0 | no complications |  |  |
| sdcN04 | numbness | sdcO10.0 | no late complications |  |  |
| sdcN05 | memory impaired | sdcO11 | potassium problems |  |  |
| sdcN06 | intermittent micturition | sdcO12 | low alat |  |  |
| sdcN07 | stomach ache | sdcO13 | metallic taste |  |  |
| sdcN08 | low of vibration sensitivity | sdcO14 | normal alat |  |  |
| sdcN09 | neuritis | sdcO15 | newly diagnosed diabetes |  |  |
| sdcN10 | neurological complications | sdcO16 | late complications |  |  |
| sdcN11 | neuropathy | sdcO17 | white coat hypertension |  |  |
| sdcN12 | neuropathogenics |  |  |  |  |
| sdcN13 | loss of vibration sensitivity |  |  |  |  |
| sdcN14 | insomnia |  |  |  |  |
| sdcN15 | arb_test |  |  |  |  |
